# Structural and Functional Insights into CP2c Transcription Factor Complexes

**DOI:** 10.3390/ijms23126369

**Published:** 2022-06-07

**Authors:** Seung Han Son, Min Young Kim, Eunbi Jo, Vladimir N. Uversky, Chul Geun Kim

**Affiliations:** 1 Department of Life Science, Research Institute for Natural Sciences, College of Natural Sciences, Hanyang University, Seoul 04763, Korea; imsangok12@naver.com (S.H.S.); 5718my@naver.com (M.Y.K.); choeunbi0324@naver.com (E.J.); 2 Department of Molecular Medicine, USF Health Byrd Alzheimer’s Research Institute, Morsani College of Medicine, University of South Florida, Tampa, FL 33612, USA; 3 CGK Biopharma Co., Ltd., 222 Wangshipri-ro, Sungdong-gu, Seoul 04763, Korea

**Keywords:** transcription factor CP2c complexes, DNA binding motifs, subcellular localization, transcriptional regulation, therapeutics

## Abstract

CP2c, also known as TFCP2, α-CP2, LSF, and LBP-1c, is a prototypic member of the transcription factor (TF) CP2 subfamily involved in diverse ubiquitous and tissue/stage-specific cellular processes and in human malignancies including cancer. Despite its importance, many fundamental regulatory mechanisms of CP2c are still unclear. Here, we uncover unprecedented structural and functional aspects of CP2c using DSP crosslinking and Western blot in addition to conventional methods. We found that a monomeric form of a CP2c homotetramer (tCP2c; [C4]) binds to the known CP2c-binding DNA motif (CNRG-N(5~6)-CNRG), whereas a dimeric form of a CP2c, CP2b, and PIAS1 heterohexamer ([C2B2P2]_2_) binds to the three consecutive CP2c half-sites or two staggered CP2c binding motifs, where the [C4] exerts a pioneering function for recruiting the [C2B2P2]_2_ to the target. All CP2c exists as a [C4], or as a [C2B2P2]_2_ or [C2B2P2]_4_ in the nucleus. Importantly, one additional cytosolic heterotetrameric CP2c and CP2a complex, ([C2A2]), exerts some homeostatic regulation of the nuclear complexes. These data indicate that these findings are essential for the transcriptional regulation of CP2c in cells within relevant timescales, providing clues not only for the transcriptional regulation mechanism by CP2c but also for future therapeutics targeting CP2c function.

## 1. Introduction

Precise spatiotemporal regulation of gene expression is essential for normal cell function and understanding of this process is central in current biology, where transcriptional regulation is a fundamental and key process of gene expression in all organisms. Transcription factors (TFs) bind DNA in a sequence-specific manner to promoters of genes, which are near their transcription start site, and to enhancers, regulatory regions that can control gene expression by physically contacting promoters through long-range interactions [1,2,3,4]. TFs work alone or with other proteins to orchestrate the transcriptional activation of a specific gene by integrating the information encoded by many regulatory control elements, namely coactivators, chromatin remodelers, histone acetyltransferases, histone deacetylases, kinases, and methylases [3,4]. However, eukaryotic TFs show a highly divergent spectrum of regulatory mechanism and function, and we do not completely know the underlying molecular mechanisms.

The transcription factor, CP2c (also known as TFCP2, α-CP2, LSF, LBP1c, UBP-1, and SEF-1), is an evolutionary conserved TF belonging to the TFCP2/GRH family [5,6,7,8,9,10]. The CP2 TF subfamily consists of six isoforms in humans (LBP-1a, -1b, -1c, -1d, -9, and -32, where the LBP-1a/b and LBP-1c/d are generated by alternative splicing) and four in mice (CP2a/NF2d9, CP2b, CP2c/Tfcp2, and Crtr1/Tfcp2l1, where CP2a/b are generated by alternative splicing) [8,10,11]. CP2c is known to exert vital functions in cell proliferation, cell cycle, and differentiation—including hematopoiesis, immune response, and neural development [7,12,13,14,15,16,17]. CP2c is important in the pathogenesis of various malignant diseases, such as human immunodeficiency virus infection and acquired immunodeficiency syndrome (HIV/AIDS), allergic response, inflammation, Alzheimer’s disease, and hemoglobinopathies [18]. Along with its main oncogene function in hepatocellular carcinoma (HCC) [19], CP2c also plays a multifaceted role in chemoresistance, angiogenesis, and epithelial–mesenchymal transition (EMT) [18,19,20,21]. The highly divergent spectrum of actions could be associated with the presence of several specific members of the TFCP2 family that are characterized by variations in the DNA binding modules, different interactomes, and specific patterns of tissue distribution [8]. However, we do not know the underlying molecular mechanisms of how this ubiquitous CP2c exerts such diverse tissue/lineage-specific regulation of gene expression and in human malignancies.

Two kinds of CP2c TF complexes, a homotetrameric CP2c complex (tCP2c) and a heterohexameric complex (CBP) containing CP2c, CP2b, and Pias1, were suggested. Initially, CP2c was reported to bind as a tCP2c to a CNRG-N(5~6)-CNRG (where N = G, A, T, or C and R = G or A) DNA motif present in diverse cellular and viral promoters [22]. Later, CP2c was discovered to bind to the α-globin promoter by forming a CBP complex [8,23]. It is proposed that the CBP could bind to DNA with two or more consecutive or overlapping CP2c binding motifs but not with a single CP2c binding motif, whereas the tCP2c binds to a single CP2c binding motif in several erythroid gene regulatory regions [24], and thus both tCP2c and CBP complexes are involved in the transcriptional activation of erythroid genes. Many questions remain, however, such as how two different CP2c complexes discriminate their DNA binding motifs and exert differential functions to their target genes, what the physiological relevance of these complexes is, and the stoichiometry of these complexes in DNA-bound and -unbound states in the nucleus.

In this work, we examined the structural and functional aspects of CP2c using a newly developed dithiobis(succinimidyl propionate) (DSP) crosslinking and Western blot (DSP XL-WB), in addition to conventional methods, uncovering several unprecedented findings about stoichiometries, DNA binding targets, and regulation of nuclear levels of CP2c TF complexes.

## 2. Results

### 2.1. Differential Binding of tCP2c and CBP to the Erythroid Gene Regulatory Regions in MEL Cells

**Figure 1:**
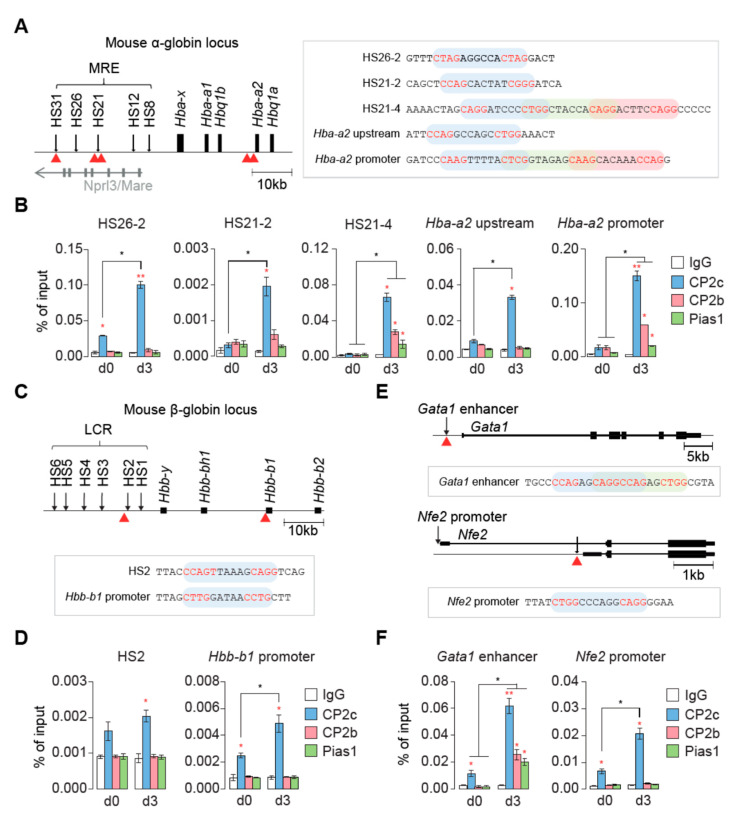
Differential binding of tCP2c and CBP to the erythroid gene regulatory regions in mouse erythroleukemia (MEL) cells. (**A**,**B**) Differential binding modes of CP2c complex proteins in the regulatory regions of the mouse α-globin locus. (**A**) Schematic representation of the mouse α-globin locus (left) and illustration of CP2c binding motifs in several regulatory regions (HS26, HS21, *Hba-a2* upstream, and *Hba-a2* promoter). (**B**) Chromatin immunoprecipitation (ChIP)-qPCR profiles of the CP2c complex proteins in uninduced (d0) and induced (differentiation day 3, d3) MEL cells. (**C**,**D**) Differential binding modes of CP2c complex proteins in the regulatory regions of the mouse β-globin locus. (**C**) Schematic representation of the mouse β-globin locus (left) and illustration of CP2c binding motifs in several regulatory regions (HS2 and *Hbb-b1* promoter). (**D**) ChIP-qPCR profiles of the CP2c complex proteins in d0 and d3 MEL cells. (**E**,**F**) Differential binding modes of CP2c complex proteins in the *Gata1* proximal enhancer and in the *Nfe2* promoter regions. (**E**) Schematic representations of the *Gata1* proximal enhancer and the *Nfe2* promoter regions, illustrating their CP2c binding motifs. (**F**) ChIP-qPCR profiles of the CP2c complex proteins in d0 and d3 MEL cells. Data are means ± SD of two independent biological replicates. Asterisks indicate significant differences (Student’s *t*-test): ** *p* < 0.01; * *p* < 0.05.

To better understand the differential binding mechanism of two different CP2c complexes, namely tCP2c and CBP, we recalled the binding status of CP2c complex proteins in some regulatory regions of erythroid genes by chromatin immunoprecipitation–quantitative PCR (ChIP-qPCR) during MEL cell differentiation in vitro. Previously, we examined the recruitment of CP2c complex proteins to the regulatory regions by focusing on the Gata1/Fog1-Mbd2/NuRD complex axis [24]. We found that all CP2c complex proteins bind to the endogenous α-globin promoter, and that the extent of binding increased in differentiated MEL cells. Here, we re-examined the binding efficiency of CP2c complex proteins focused on the configuration of CP2c binding motifs (Figure 1). In the mouse α-globin locus, all CBP proteins (CP2c, CP2b, and Pias1) were recruited to the MRE HS21-4 (the fourth fragment in the hypersensitive site 21 region of the α-globin major regulatory element) and to the *Hba-a2* promoter, having four consecutive CP2c half-sites (CNRG separated by five or six nucleotides) of a CP2c binding motif (Figure 1A,B).

On the contrary, only CP2c was recruited to the α-globin MRE HS26-2 and HS21-2, the *Hba-a2* upstream promoter, the mouse β-globin LCR HS2, the *Hbb-b1* promoter, and the *Nfe2* promoter, where two consecutive CP2c half-sites (i.e., a single CP2c binding motif (CNRG-N(5~6)-CNR(G/C))) existed (Figure 1A–F). Surprisingly, all CP2c complex proteins were recruited to the *Gata1* enhancer where two overlapped CP2c binding motifs staggered by four base pairs existed (Figure 1E,F). These data suggest that bindings of tCP2c and CBP are discriminated by the number and configuration of CP2c binding motifs (or CP2c half-sites), and thus a single CP2c binding motif (with two consecutive CP2c half-sites) is sufficient for tCP2c binding, whereas additional copies of CP2c binding motifs (i.e., consecutive three or more CP2c half-sites or two staggered CP2c binding motifs) are required for CBP binding. It is of note here that substantial recruitment of CBP occurred during differentiation in the *Gata1* enhancer, where tCP2c was preoccupied in undifferentiated cells (Figure 1F). Since preoccupation of tCP2c, but not CBP, was significant in undifferentiated cells regardless of the number of consecutive CP2c half-sites (Figure 1B,D,F), there might exist an unknown mechanism for differentiated cell-specific recruitment of CBP (see below).

### 2.2. The tCP2c Binds to Sequences Containing Two Consecutive CP2c Half-Sites, Whereas the CBP Binds to Sequences Containing Three Consecutive CP2c Half-Sites or Two Staggered CP2c Binding Motifs

**Figure 2:**
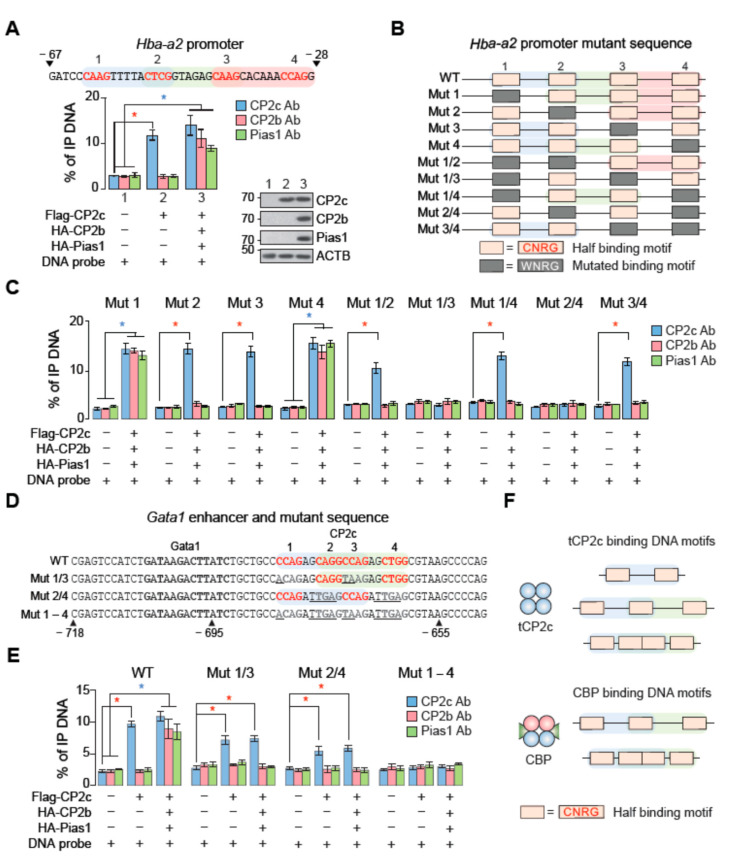
tCP2c binds to sequences containing two consecutive CP2c half-sites, whereas CBP binds to sequences containing three consecutive CP2c half-sites or two staggered CP2c binding motifs. (**A**–**C**) Dissection of minimal CP2c binding motifs for the binding of tCP2c and CBP complexes in the *Hba-a2* promoter. (**A**) CP2c binding motifs in the *Hba-a2* promoter (top) and DIP profiles showing the binding capability of both tCP2c and CBP complexes to the CP2c binding motifs in the *Hba-a2* promoter (bottom). To validate where the DIP signals were originated from when the probe bound to the immunoprecipitated proteins, Western blots were performed using specific antibodies against CP2c, CP2b, and Pias1 (shown at the right of bottom). (**B**) Schematic diagram of probes with wild type or mutated CP2c binding motifs in combination. (**C**) DIP profiles showing differential binding of tCP2c and CBP complexes on the probes with various combinations of the CP2c binding motif mutation. (**D**,**E**) Dissection of minimal CP2c binding motifs for the binding of tCP2c and CBP complexes in the *Gata1* enhancer. (**D**) CP2c binding motifs in the *Gata1* enhancer region and schematic diagram of probes with wild type or mutated CP2c binding motifs in combinations. (**E**) DIP profiles showing differential binding of tCP2c and CBP complexes on the probes with various combinations of the CP2c binding motif mutations. Data are means ± standard deviations (SD) of two independent biological replicates. Asterisks indicate significant differences (Student’s *t*-test): * *p* < 0.05. (**F**) Schematic presentations of two different CP2c transcriptional complexes and their DNA binding motifs.

To clarify the differential binding modes of tCP2c and CBP to the various configurations of CP2c binding motifs, we tested the binding abilities of CP2c complex proteins by in vitro DNA co-immunoprecipitation (DIP) assays [25]. DIP could measure and quantify the amounts of eluted DNA probes co-immunoprecipitated by a specific antibody against a target protein if specific DNA–protein complexes existed in the reaction mixture containing nuclear extracts and the radiolabeled DNA probe. When the *Hba-a2* promoter probe containing four consecutive CP2c half-sites was reacted with 293T nuclear extracts overexpressing epitope-tagged CP2c protein alone or CP2c, CP2b, and Pias1 all together, both CP2c by itself and CP2c/CP2b/Pias1 together could bind to the probe (Figure 2A), suggesting both tCP2c and CBP bind to the DNA containing four consecutive CP2c half-sites. Next, when we tested a series of *Hba-a2* promoter mutants containing various CP2c half-site mutations in combination (Figure 2B), we found that more than two or three consecutive CP2c half-sites were needed for tCP2c and CBP bindings, respectively (Figure 2C). In the case of *Gata1* enhancer mutants (Figure 2D), two staggered CP2c binding motifs were required for CBP binding, whereas each of two consecutive CP2c half-sites was sufficient for tCP2c binding (Figure 2E). These data indicate that the CBP binds to DNA with two different configurations of CP2c binding motifs, i.e., three consecutive CP2c half-sites or two staggered CP2c binding motifs, whereas the tCP2c binds only to DNA with two consecutive CP2c half-sites.

### 2.3. Stable DNA Binding Occurs Either by a Monomeric tCP2c or by a Dimeric CBP

**Figure 3:**
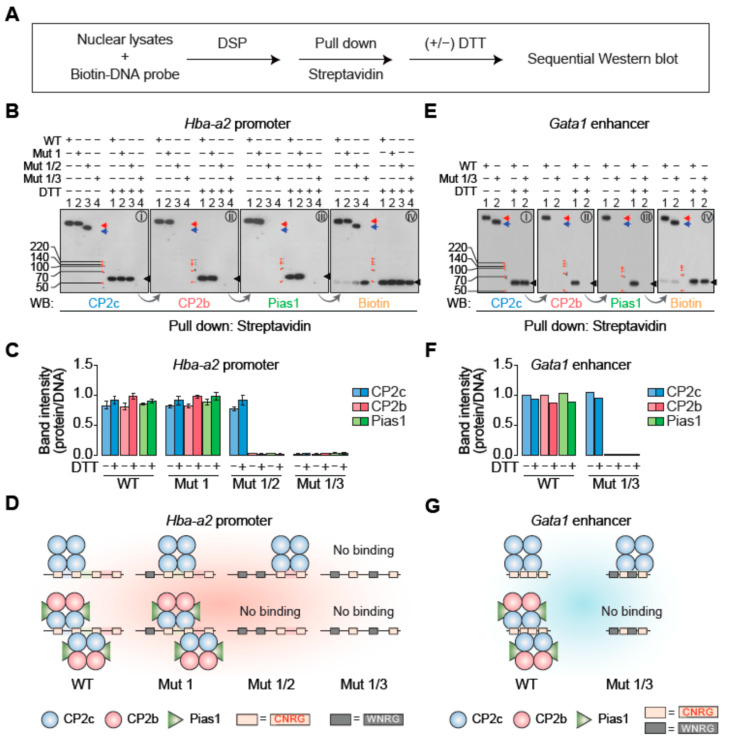
Stable DNA binding occurs either by monomeric tCP2c ([C4]) or dimeric CBP ([C2B2P2]_2_). (**A**) Experimental scheme for the determination of identity and stoichiometry of CP2c complex proteins by DSP crosslinking and sequential Western blot. (**B**–**D**) Identity and stoichiometry of CP2c complex proteins bound to the wild type or various CP2c binding motif mutations in the *Hba-a2* promoter (see Figure 2B). Immunoblots (**B**) and the quantification of each spot (**C**) show that the same relative amounts of CP2c, CP2b, and Pias1 exist in the CBP complex, while both tCP2c and CBP complexes contain the same amounts of CP2c. Samples were subjected to 10% SDS-PAGE. N = 2. (**D**) Schematic models showing stoichiometry of tCP2c and CBP complexes bound to their wild type or various CP2c binding motif mutants in the *Hba-a2* promoter. (**E**–**G**) Identity and stoichiometry of CP2c complex proteins bound to the wild type or various CP2c binding motif mutations in the *Gata1* enhancer (see Figure 2D). Immunoblots (**E**) and the quantification of each spot (**F**) show that the same relative amounts of CP2c, CP2b, and Pias1 exist in the CBP complex, while both tCP2c and CBP complexes contain the same relative amounts of CP2c. Samples were subjected to 10% SDS-PAGE. (**G**) Schematic models showing stoichiometry of tCP2c and CBP complexes bound to their wild type or mutant CP2c binding motif in the *Gata1* enhancer.

To identify CP2c complexes bound to the target DNA and estimate their stoichiometry, we employed a method using chemical (DSP) crosslinking and Western blotting (DSP XL-WB) (Figure 3A). Since DSP is a disulfide-bond-containing cross-linking reagent and can be chemically cleft by reducing reagent treatment, we could identify components in the crosslinked complex and estimate their stoichiometry when applied to XL-WB. To discriminate the binding of tCP2c and CBP to the probes containing various numbers of consecutive CP2c half-sites, we used biotin-labeled probes of the wild type (WT) *Hba-a2* promoter and the selected Mut 1, Mut 1/2, and Mut 1/3 mutants (containing four, three, or two consecutive, or single CP2c half-sites, respectively; see Figure 2B) in the reaction containing 293T nuclear extracts. Assuming all the CBP complexes have the same relative amounts of CP2c, CP2b, and Pias1 [23], the complex composition was estimated by measuring relative band intensities in a sequential Western blot by repeating steps of washing out the probe and reprobing. To obtain a relative value for each complex band, the intensity value measured without DTT treatment was divided by the intensity of the monomer band obtained by DTT treatment. WB analyses of the complexes pulled down with biotin-labeled DNA (using streptavidin bead) identified the same relative amounts of all CP2c complex proteins in the complexes formed with the WT and Mut 1 probes, whereas only CP2c was found in the complex with Mut 1/2, and no protein at all was bound to Mut 1/3 (Figure 3B). Our finding of the same relative amounts of CP2c, CP2b, and Pias1 in the complexes with WT and Mut 1 probes was not different when analyzed after DTT treatment (Figure 3C), and it was consistent with our previous report regarding the existence of the heterohexamer complex containing two units each of CP2c, CP2b, and Pias1 [23]. Here, Pias1 serves as a clamp between two CP2 proteins, while CP2c binds directly to the target DNA and CP2b mediates strong transactivation. Importantly, the relative amounts of CP2c in the CBP complexes formed with the WT and Mut 1 probes were not different from those in the tCP2c (i.e., [C4]) complex with the Mut 1/2 probe (Figure 3B,C), suggesting dimeric CBP complexes (i.e., [C2B2P2]_2_) bind to the DNA containing three consecutive CP2c half-sites, since tCP2c consists of four CP2cs [22]. Similarly, the same relative amounts of all CP2c complex proteins existed in the complexes with the WT *Gata1* enhancer probe containing two staggered CP2c binding motifs, and the amounts of CP2c in the WT probe were not different from those in the Mut 1/3 probe containing a single CP2c binding motif (Figure 3D,E). Taken together, our data indicate that the [C2B2P2]_2_ binds to the DNA containing either three consecutive CP2c half-sites or two staggered CP2c binding motifs, whereas the [C4] binds to the DNA containing two CP2c consecutive half-sites (or one CP2c binding motif) (Figure 3F). It is important to note here that [C2B2P2]_2_, but not [C4], was bound to the probes containing three or four consecutive CP2c half-sites where both [C4] and [C2B2P2]_2_ could theoretically bind.

### 2.4. CP2c Exists in the Nucleus as a Complex of Either Monomeric tCP2c or Multimeric CBPs

**Figure 4:**
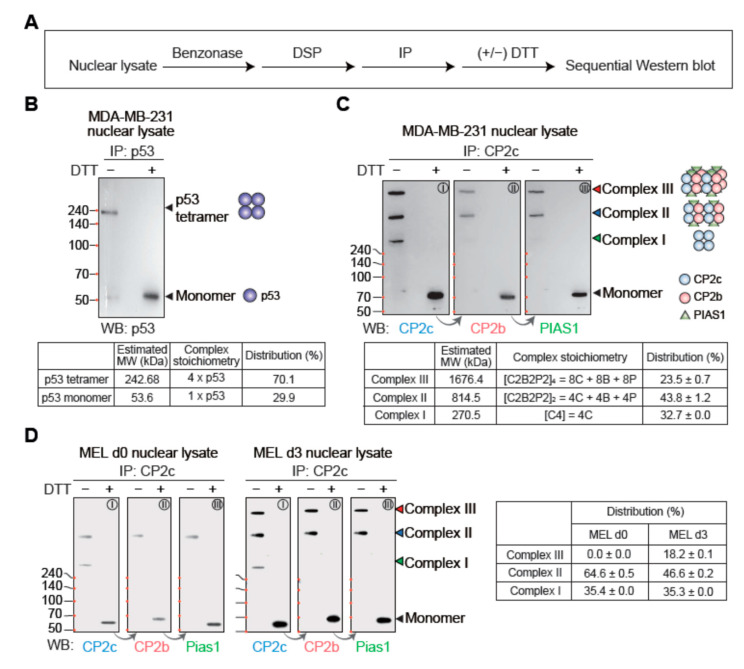
CP2c exists as complexes of either monomeric tCP2c or multimeric CBPs in the nucleus. (**A**) Experimental scheme to identify CP2c complex types and proportions of each complex in the nucleus by DSP crosslinking and sequential Western blot. (**B**) Immunoblots showing preferential distribution of p53 tetramer over monomer in the nucleus (top) and estimation of the protein complex sizes, and stoichiometry and relative ratios of each complex (bottom). Samples were subjected to 10% SDS-PAGE. The protein complex sizes were estimated by determining the relative migration distance [27] of the protein standards. (**C**,**D**) Identification of various nuclear forms of tCP2c or CBP complexes and estimation of the protein complex sizes, and stoichiometry and relative ratios of each complex. Nuclear extracts of MDAMB-231 (**C**) and MEL cells during in vitro differentiation (d0 and d3) (**D**) were used for sequential Western blotting. Samples were subjected to 10% SDS-PAGE.

As CP2c solely exists as either a monomeric [C4] or a [C2B2P2]_2_ in a DNA-bound state, we wondered about the DNA-unbound CP2c states in the nucleus. CP2c was initially identified as a dimer in solution by sedimentation analyses. However, a tetrameric form of CP2c was later observed by Western blot after chemical crosslinking [22]. In addition, the existence of another form of CP2c complex, CBP, was proposed [23]. Therefore, it is still unclear how many kinds of nuclear CP2c complexes exist in solution, and what the stoichiometry of each complex is. We exploited DSP XL-WB assays to identify types of complexes and estimate their stoichiometry and proportions in cells, i.e., we performed sequential Western blotting of proteins immunoprecipitated with specific antibodies to the DSP-crosslinked nuclear extracts (Figure 4A). To obtain a relative value for each complex band, the intensity value measured without DTT treatment was divided by the intensity of the monomer band obtained by DTT treatment. Relative band intensities in a Western blot were used for the estimation of the complex composition, assuming that all of the complexes have the same relative amounts of the different proteins. After obtaining each relative quantitative value from the results of IP and blotting with different antibodies, data were presented as mean ± standard error. The relative amount of the complex was calculated based on the theoretical number of components (CP2a, CP2b, CP2c, and PIAS1) in each complex and used for the estimation of ratios of each complex in cells. The usefulness of this protocol was validated by analyzing the oligomerization status of the p53 tumor suppressor (Figure 4B), which has been known to exist mainly as a homotetramer with a minority of monomer in cells [26].

Importantly, a free state of CP2c (i.e., a monomeric or dimeric CP2c) was barely found and, instead, three kinds of CP2c complexes, complex I, II, and III, were identified in MDA-MB-231 cell nuclear extracts (Figure 4C). The protein band intensity analysis in the immunoblots suggested a 1.8- and 1.9-fold higher content of CP2c in complexes II and III compared with complex I, comprising more than 5.5-fold higher amounts of [C2B2P2]s over [C4] (Figure 4C). Since CBP might be consisted of equimolar amounts of CP2c, CP2b, and Pias1, as revealed by determination of the interfaces of each protein important for complex formation and their functional relevance [23], the estimated size and stoichiometry of each complex suggested that complex I was [C4], whilst complexes II and III were [C2B2P2]_2_ and [C2B2P2]_4_, respectively. Importantly, the same three types of CP2c complexes were also identified in the DSP XL-WB assays in MEL cell nuclear extracts (Figure 4D). CP2c complexes I, II, and III appeared in erythroid differentiated (d3) MEL cells, whereas only complexes I and II existed in the undifferentiated (d0) MEL cells (Figure 4D). Since cellular levels of CP2c and CP2b, but not Pias1, are known to increase two- to three-fold during in vitro MEL cell differentiation [24], these data suggest that complex III ([C2B2P2]_4_) appears only when sufficiently high levels of nuclear CP2c and CP2b are attained. Moreover, since complexes I and II, but not complex III, were engaged in CP2c-target DNA binding (see Figure 3D,G), complex III is suggested to be a nuclear reservoir of CP2c complexes. Taken together, our data suggest that there are three kinds of CP2c complexes in nucleus, where complexes I and II ([C4] and [C2B2P2]_2_) engage in CP2c-target DNA binding and complex III ([C2B2P2]_4_) functions as a nuclear reservoir of CP2c complexes. However, it is of note here that since crosslinked protein complexes do not migrate according to molecular weight standards, and the molecular weight standards are only in the range of 50~240 kDa; our estimation of the mass of crosslinked proteins may have inherent caveats to be proven by other appropriate methods.

### 2.5. Cytosolic CP2a Regulates Subcellular Distribution and Dynamics of Various CP2c Complexes

**Figure 5:**
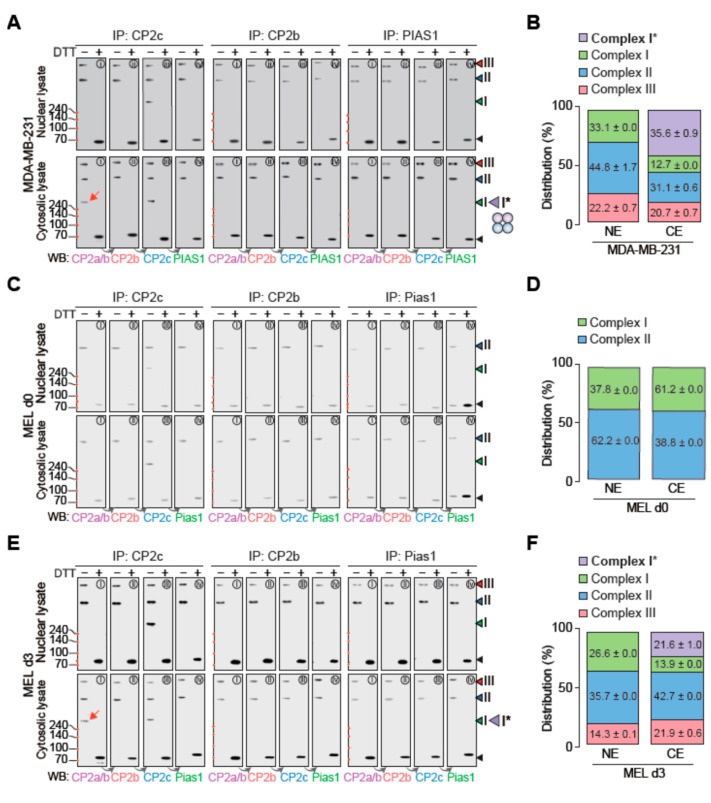
Cytosolic CP2a regulates the subcellular distribution and dynamics of various CP2c complexes. (**A**,**B**) CP2c complex types and subcellular distribution of each complex in MDA-MB-231 cells by DSP crosslinking and sequential Western blot. (**A**) Immunoblots showing various nuclear (top) and cytosolic (bottom) CP2c complexes. (**B**) Estimated fractions of each CP2c complex type in the nucleus and cytosol. (**C**–**F**) Subcellular distribution and dynamics of various CP2c complexes in HMBA-induced differentiating MEL cells in vitro. Immunoblots showing various nuclear (top) and cytosolic (bottom) CP2c complexes in d0 (**C**) and d3 (**E**) MEL cells. Estimated fractions of each CP2c complex type are shown in the nucleus and cytosol in d0 (**D**) and d3 (**F**) MEL cells. Samples were subjected to 10% SDS-PAGE. Red arrows in the immunoblots highlight the existence of CP2a in the cytosol-specific CP2c complex, complex I* ([C2A2]). Note, that to evaluate the ratios of each complex in the nucleus and cytosol, we averaged the values estimated from the Western blots that were obtained from IPs with CP2c and CP2b, excluding PIAS1 (or Pias1), since we thought that the PIAS1-specific Ab used for IP was saturated in our assay. We found a free form of Pias1 in the sample of MEL cells at d0, but not in other samples, where we used the same amounts of PIAS1 Ab for IPs. Because PIAS1 (or Pias1) could exist in various forms beyond the CBP complex, we expected to find a free PIAS1 (or Pias1) form in all experiments, if not limited by the amounts of the PIAS1 Ab.

It is known that two alternative splicing variants, CP2a and CP2b, exclusively localized in the cytosol and nucleus, respectively [16]. Here, the CP2b-specific exon oversees the nuclear localization of CP2b and, depending on the relative levels of CP2a and CP2b, CP2c can be found either in the nucleus or cytosol, with CP2c being intrinsically localized in the cytosol in the absence of CP2a/CP2b. However, it is important to note that this finding was based on the subcellular localization of the ectopically overexpressed factor, but not the endogenous one. Accordingly, we re-examined the subcellular distribution of endogenous CBP complex proteins by Western blot after fractionation of proteins into nuclear lysate and cytosolic lysate (Figure S1). Importantly, CP2b existed in both the nucleus and cytosol. Since Lamin B1, which is a nuclear protein present only in the nucleus in this assay, we concluded that based on the results of our fractionation protocol of proteins into the nuclear and cytosolic extracts, it would be pertinent to say that CP2b exists in both the nucleus and cytosol. Taking these observations into account, we performed DSP XL-WB assays to see the subcellular distribution and dynamics of CP2c complexes in connection with cytosol-specific CP2a. To control and minimize variations for our estimation, the final amounts of nuclear extract and cytosolic extract that were loaded onto the gel for analysis came from the same number of cells, and the exposure times were consistent. Firstly, we analyzed CP2c complexes in the nuclear and cytosolic extracts of MDA-MB-231 cells by DSP XL-WB (Figure 5A).

Since CP2b differs from CP2a by only one additional exon sequence [8], we used two different antibodies, one detecting both CP2a and CP2b, and the other detecting only CP2b, in order to discriminate between CP2a and CP2b. In addition, to determine the possibility of additional CP2c-free protein complexes containing other CP2c complex proteins, we performed additional IPs with CP2b- and PIAS1-specific antibodies. The nuclear CP2c complexes analyzed by IP with CP2b and PIAS1 antibodies were not different from those with the CP2c antibody, exhibiting complexes I, II, and III (Figure 5A, upper panels). However, in addition to the same three complexes that appeared in the nucleus, one more additional complex (complex I* [C2A2], which might consist of two each of CP2c and CP2a, when estimating the relative amounts of CP2a and CP2c in the complex I/I* in relation to those of complexes II and III in the same blots) was observed in the cytosol (Figure 5A, lower panels). The existence of the cytosol-specific complex I* is not surprising since CP2a solely appeared in the cytosol due to the lack of a nuclear localization signal, although it had structural similarity to CP2c or CP2b and the recombinant CP2a showed DNA binding ability alone or in combination with CP2c or CP2b in vitro [8,16]. It is important to note here that, when compared with the nuclear fractions of the complexes, the cytosolic fraction of complex I ([C4]) is greatly reduced as complex I* ([C2A2] is increased (Figure 5B), suggesting that, by forming complex I*, CP2a functions to control the levels of nuclear CP2c complexes to cope with cellular demands.

The cytosol-specific complex I* was also observed in d3 MEL cells (Figure 5C–F). In d0 cells, only complexes I and II were detected both in the nucleus and cytosol with a similar ratio of complexes, yet complex II was more prominent (Figure 5C,D). In d3 cells, in addition to complexes I and II, complex III appeared in both the nucleus and cytosol as cellular CP2c and CP2b levels were increased (Figure 5E). These data suggest that CP2c complexes are assembled in the cytosol before moving into the nucleus, and the amounts of CP2c complexes are increased according to higher levels of component proteins by differentiation. Importantly, an additional complex, complex I*, was observed in the cytosol of d3 cells as a decrease in the relative proportion of cytosolic complex I to nuclear complex I was observed (Figure 5F). Since the cytosolic complex I* appeared only in differentiated cells, we speculated that by forming complex I*, CP2a functions in the cytosol to control the levels of other CP2c complexes ([C4], [C2B2P2]_2_, and [C2B2P2]_4_) to cope with cellular demands.

**Figure 6:**
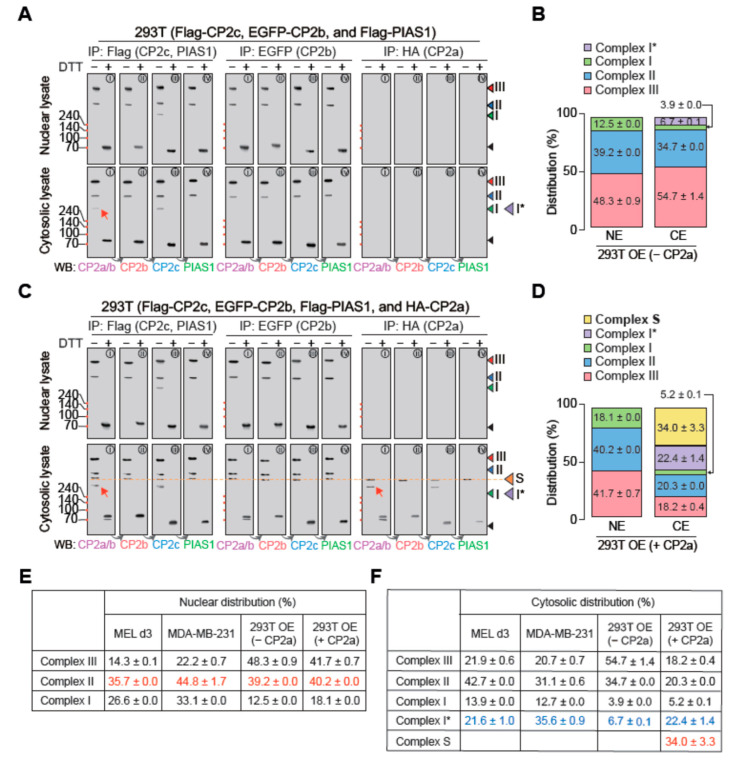
Subcellular distribution and dynamics of various CP2c complexes in cells with overexpression of CP2c complex proteins. (**A**,**B**) CP2c complex types and subcellular distribution of each complex in 293T cells where epitope tagged CP2c complex proteins (Flag-CP2c, EGFP-CP2b, and Flag-PIAS1) were ectopically overexpressed. (**C**,**D**) CP2c complex types and subcellular distribution of each complex in 293T cells where epitope tagged CP2c complex proteins (Flag-CP2c, EGFP-CP2b, and Flag-PIAS1) and CP2a were ectopically overexpressed. Immunoblots (**A**,**C**) showing various CP2c complexes in the nucleus (top) and cytosol (bottom) and the quantified distribution plots of each complex in the nucleus and cytosol (**B**,**D**). Red arrows in the immunoblots highlight the existence of CP2a in the cytosol-specific CP2c complex, complex I* ([C2A2]). The red dotted line indicates another cytosolic CP2c complex, complex S that appeared in cells by ectopic overexpression of CP2c complex proteins. Samples were subjected to 10% SDS-PAGE. (**E**,**F**) Estimated ratios of each CP2c complex in the nucleus (**C**) and cytosol (**D**) in comparison with those of d3 MEL and MDA-MB-231 cells.

To test whether CP2c complexes could be maintained as a steady state in the nucleus by the regulatory function of CP2a in the cytosol, we perturbed cellular levels of CP2c complex proteins in the presence or absence of ectopic overexpression of CP2a in 293T cells and then analyzed the nuclear and cytosolic CP2c complexes (Figure 6A–D).

When we quantified each complex in cells with ectopic overexpression of CP2c complex proteins in the presence or absence of ectopic overexpression of CP2a, the complexity and abundancy of nuclear CP2c complexes was similar in both groups, although those in cytosol were quite different (Figure 6B,D). In addition to complex I*, another CP2a-containing complex, complex S, was observed in the cytosol only when CP2a was ectopically overexpressed. Importantly, among nuclear complexes, the relative amounts of complex II, comprising to [C2B2P2]_2_, were quite similar in all cell types used in this study, including MDA-MB-231 and d3 MEL cells (Figure 6E), whereas the relative amounts of each cytosolic complex, including complex I* and complex S, were quite variable among cell types (Figure 6F). These data support the notion that CP2a forms complexes (complex I* and then complex S) with the surplus CP2c-complex proteins in the cytosol to maintain the nuclear levels of complexes (specifically complex II) needed to cope with the cellular demands, primarily controlling the nuclear [C4] levels.

### 2.6. tCP2c Exerts a Pioneering Function for Recruiting [C2B2P2]_2_ to the CP2c Binding Sites with Three or More CP2c Half-Sites

Since tCP2c requires one CP2c binding motif (two consecutive CP2c half-sites) for DNA binding, tCP2c could also bind to the CBP binding sequences of constitutive three CP2c half-sites or two staggered CP2c binding motifs (Figure 2F). However, we found that [C2B2P2]_2_, but not [C4], was bound to the *Hba-a2* WT and Mut 1, and the *Gata1* enhancer probes in vitro (Figure 3D,G). Moreover, the tCP2c was preoccupied to the *Gata1* enhancer sequences of two staggered CP2c binding motifs in undifferentiated MEL cells, whereas significant enhancement of CBP binding occurred in differentiated cells (Figure 1F). These data indicate that the efficiency of [C4] and [C2B2P2]_2_ binding to the CP2c target DNA sequences with constitutive three CP2c half-sites or two staggered CP2c binding motifs is enigmatic, and thus there might be a subtle discrimination mechanism for [C4] and [C2B2P2]_2_ in target binding. In addition, since a majority of CP2c existed predominantly as multimeric CBPs (for example, only 1/12 of nuclear CP2c is in [C4]; Figure 4), the nuclear abundance of CP2c complexes should be accounted for in this binding discrimination mechanism.

**Figure 7:**
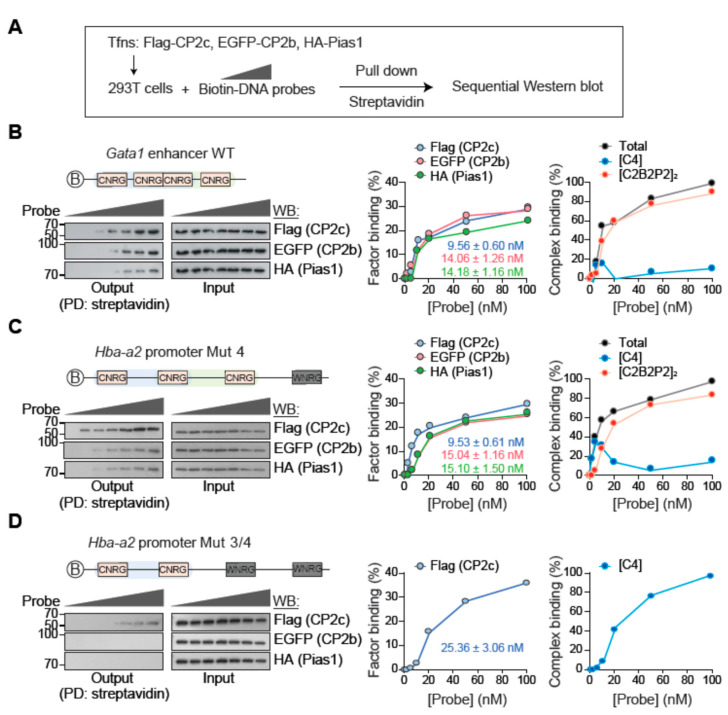
tCP2c exerts a pioneering function for recruiting [C2B2P2]_2_ to the CP2c binding sites with three or more CP2c half-sites. (**A**) Experimental scheme to test binding efficiency of CP2c complex proteins in vitro by Western blot analysis. (**B**–**D**) Immunoblots and estimated dissociation constant (Kd) and DNA binding efficiency of [C4] and [C2B2P2]_2_ in probes containing the *Gata1* enhancer (**B**), the *Hba-a2* promoter Mut 4 (**C**), and the *Hba-a2* promoter Mut 3/4 (**D**). See Figure S1 for additional data for binding efficiency to other biotin-labeled probes.

To determine the binding efficiency of [C4] and [C2B2P2]_2_, we tested binding affinities of [C4] and [C2B2P2]_2_ to the DNA probes containing various numbers of CP2c half-sites by DIP using biotin-tagged probes and epitope-tagged factors overexpressing 293T nuclear extracts (Figure 7A and Figure S2A). Since [C4] consisted solely of CP2c, whereas [C2B2P2]_2_ contained CP2b and Pias1 in addition to CP2c, the DNA binding affinities of [C4] and [C2B2P2]_2_ were estimated by measuring the CP2c and the averaged CP2b and Pias1 binding efficiency. In the *Gata1* enhancer probe (having two staggered CP2c binding motifs), although CP2c (Kd of 9.56 ± 0.60 nM) showed a 1.5-fold higher binding affinity than CP2b or Pias1 (14.06 ± 1.26 nM and 14.18 ± 1.16 nM), [C2B2P2]_2_ surpassed [C4] by increasing probe concentration (Figure 7B). In addition, the same phenomenon occurred in the test using *Hba-a2* promoter probes. In the *Hba-a2* promoter Mut 4 probe containing three consecutive CP2c half-sites, CP2c (Kd of 9.53 ± 0.61 nM) showed a 1.5-fold higher binding affinity than CP2b or Pias1 (15.04 ± 1.16 nM and 15.10 ± 1.50 nM), but [C2B2P2]_2_ also surpassed [C4] by increasing probe concentration (Figure 7C). This phenomenon was recapitulated in the tests using a WT *Hba-a2* promoter (having four consecutive CP2c half-sites) or a *Hba-a2* Mut 1 (having three consecutive half-sites) probe, although the binding affinities of CP2c and CP2b (or Pias1) were somewhat varied, depending on the probe contexts (Figure S2B,C). Therefore, these data suggest that [C4] firstly binds to the sequences, owing to its high binding affinity, and then [C2B2P2]_2_ replaces [C4] by increasing the probe concentration, owing to its prevalence in the nucleus.

tCP2c binding affinity for the *Hba-a2* promoter Mut 3/4 probe (which had two CP2c consecutive half-sites and thus prevented [C2B2P2]_2_ binding) (25.36 ± 3.06 nM) was greatly lower (Figure 7D) than that for the probes of *Hba-a2* promoter Mut 4 and Mut 1 (from 9.53 to 10.55 nM) (Figure 7C and Figure S2C). Similarly, lower [C4] binding affinities were observed for the probes of *Hba-a2* promoter Mut 1/4 and Mut 1/2, where two consecutive CP2c half-sites existed (Figure S2D,E). Since a monomeric tCP2c is expected to bind to the probes of *Hba-a2* promoter Mut 4 and Mut 1 (Figure 2C), these data suggest that there is also synergism in tCP2c binding to the target sequences with three consecutive CP2c half-sites.

This kind of synergistic CP2c binding (Kd of 4.17 ± 0.16 nM) also occurred in the WT *Hba-a2* promoter probe with four CP2c consecutive half-sites, where tCP2c could bind two different ways and only two tCP2cs could simultaneously bind the target at maximum (Figure S1). Moreover, tCP2c occupied about 50% of the target DNA (at maximum) at high concentrations of the probe, suggesting [C4]–[C4] interaction in DNA binding is in competition to [C2B2P2]_2_. Therefore, these data suggest that there are synergisms between [C4] and [C4] and between [C4] and [C2B2P2]_2_ for target DNA binding in the context of the available amounts of nuclear CP2c complexes.

**Figure 8:**
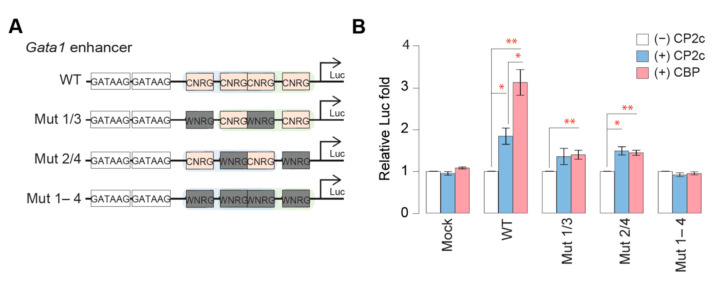
[C2B2P2]_2_ exhibits stronger transcriptional activation activity than [C4]. Luc reporter constructs containing wild type or various CP2c binding motif mutations in the *Gata1* enhancer (**A**) and their Luc reporter activities in 293T cells (**B**). To monitor the effects of [C4] and [C2B2P2]_2_ to each Luc reporter, a *CP2c* gene alone or genes encoding all the CBP complex proteins were transiently transfected. Data (means ± SD) were analyzed using Kruskal–Wallis test with Dunn’s multiple comparison post hoc test. ** *p* < 0.01 and * *p* < 0.05.

Moreover, [C2B2P2]_2_ showed superior transcriptional activation activity than [C4]. To test the transcriptional activation activity of [C4] and [C2B2P2]_2_, a luciferase assay was employed using a reporter gene under the control of the WT or various point mutations in the CNRG sequences of the *Gata1* enhancer (Figure 8A). To monitor the effects of [C4] and [C2B2P2]_2_ to each Luc reporter, a *TFCP2* gene alone or genes encoding all of the CBP complex proteins together were transiently transfected into 293T cells. [C2B2P2]_2_ showed around two-fold higher transcriptional activation activity than [C4] in the wild type *Gata1* enhancer where two [C4]s or [C2B2P2]_2_ could bind, whereas the wild type *Gata1* enhancer showed more than five-fold higher activity than Mut 1/3 or Mut 2/4 where monomeric [C4] could bind (Figure 8B). These data suggest that [C2B2P2]_2_ possesses superior transcriptional activation activity than [C4], possibly due to CP2b, which has stronger transactivation domains than CP2c, although it has a weak intrinsic DNA binding activity [8].

## 3. Discussion

Here, we examined structural and functional aspects of CP2c TF complexes in depth by employing the DSP XL-WB protocol and revealed several unprecedented facts providing invaluable insights for the transcriptional regulation of CP2c. This includes the diversity of CP2c isoforms involved in transactivation, DNA binding and heteromerization, control of subcellular localization, and interaction with specific partner proteins, such as Pias1, in addition to changes in expression levels. We found that tCP2c binds to DNA sequences containing two consecutive CP2c half-sites, whereas CBP binds to sequences containing three consecutive CP2c half-sites or two staggered CP2c binding motifs, where stable DNA binding occurs either by monomeric tCP2c ([C4]) or by dimeric CBP ([C2B2P2]_2_) in vitro. In addition, we found that CP2c always existed as complexes in cells, either as [C4] or various heteromers with other proteins (TFCP2 family proteins CP2a, CP2b, and Pias1), such as [C2B2P2]_2_ or [C2B2P2]_4_, and heterotetrameric [C2A2]. Although CP2c homodimers and homotetramers have been proposed by biochemical analysis of purified or recombinant proteins [22,28], only [C4] was observed in cell extracts by our DSP XL-WB protocol. In addition, instead of theoretical heterohexameric CBP proposed by in vitro complex forming analyses of the various mutant proteins [23], only [C2B2P2]_2_ and [C2B2P2]_4_ were observed in cells. Although detailed three-dimensional structures of these complexes must be resolved to understand the underlying mechanisms for the formation of discrete CP2c complex forms and for complex-dependent target sequence selection, our data provides an important layer of insights into the complexity of eukaryotic gene regulation.

Furthermore, in accordance with the previous report that the cytosol-specific CP2a binds to and holds CP2c in the cytosol to prevent nuclear translocation [8,16], [C2A2] only appeared in the cytosol. Since tCP2c and multimeric CBPs appear in both the cytosol and the nucleus, it is suggested that these complexes initially assemble in the cytosol immediately after completion of protein synthesis and then translocate into nucleus. We found that that net concentrations of nuclear CP2c complexes were not much different, whereas their net cytosolic concentrations were quite variable (Figure 5 and Figure 6), suggesting there were quality control mechanisms regulating the nuclear import of these complexes. Importantly, in the cytosol, [C2A2] concentration was quite variable among cell lines, whereas the net concentration of tCP2c and multimeric CBPs was evenly maintained. Moreover, net concentration of nuclear tCP2c and multimeric CBPs was maintained to the level of normal cells, although cytosolic [C2A2] and aberrant complex, Complex S, were greatly increased (Figure 5 and Figure 6). Moreover, by ectopic overexpression of CBP complex proteins irrespective of CP2a overexpression in 293T cells, those of complexes I and III were down- or up-regulated, respectively, whereas net concentrations of nuclear CP2c complex II were not much different when compared with those in MDA-MB-231 and d3 MEL cells (Figure 6). However, consistent with other data, the cytosolic concentrations of complexes, including cytosol-specific complexes I* and S, showed additional perturbation by CP2a ectopic overexpression. Therefore, we propose a model in which CP2a regulates the nuclear concentration of CP2c complexes to cope with cellular demands by squelching CP2c into [C2A2] in the cytosol at physiological conditions, or by squelching all proteins together into an aberrant heterodecameric complex at non-physiological conditions, such as ectopic overexpression of CP2c complex proteins.

We found that [C4] exerts a pioneering function for recruiting [C2B2P2]_2_ to the CP2c binding sites with three or more CP2c half-sites, concomitantly inducing synergistic binding of [C4] to the two nearby CP2c half sites. Since [C2B2P2]_2_ could exert stronger transcriptional activation activity via the CP2b-specific transcriptional activation domain and could integrate more versatile and dynamic cellular signaling by protein modifications or protein–protein interactions via Pias1 than [C4] consisting solely of CP2c, this kind of complementation between [C4] and [C2B2P2]_2_ might be a novel mechanism for efficient transcriptional regulation to cope with biological demands. In addition, the existence of nuclear [C2B2P2]_4_ suggests a facilitated intersegmental transfer mechanism between two CP2c binding sites, where a DNA-bound [C2B2P2]_4_ interacts with another DNA target, nearby or in proximal localization within a nuclear condensate, via protein–DNA interaction, forming a DNA loop or an inter-strand joining required for efficient transcriptional activation of the specific gene [29,30,31,32].

The DNA binding mode of [C2B2P2]_2_ is distinguished from that of [C4] and the evolutionary diverged GRH1 (NTF-1). The TFCP2/GRH TF family is characterized by the possession of a distinctive DNA-binding domain that bears no clear relationship to other known DNA-binding domains, except the p53 DNA-binding domain [6,33,34]. Although the GRH1 dimer is known to bind to the DNA sequence (A/T)C(A/C/T)(G/T)GTT(C/G/T), similar to an CP2c half-site, whereas tCP2c binds to a CNRG-N(5~6)-CNR(G/C) (R = [A, G], N = [G, A, C, T]) motif [35], GRH1 could not only bind a CP2c-binding motif but also an CP2c half-site, whereas CP2c is unable to stably interact with a GRH1-binding site [22], suggesting that stable DNA binding requires a CP2c half-site for the GRH1 dimer, and two consecutive CP2c half-sites for tCP2c. A pair of CP2c dimers is expected to bind to the directly repeated CP2c half-sites on the same face of DNA by requiring strict linker sequences of 5~6 base pairs, although the exact binding structure has not been investigated yet. Of note, although p53 binds to sequences comprising two copies of the sequence RRR**CATG**YYY (R = [A,G], Y = [C,T]) as a tetramer like CP2c, p53 could bind to a p53 half-site like GRH1 and spaced sequences between CATG are flexible enough to allow 0–13 base pairs through [36,37,38], suggesting that binding modes of CP2c, GRH1, and p53 are quite conserved but diverged in detail. However, in contrast to GRH1 and p53, CP2c forms other complexes of [C2B2P2]_2_ and [C2B2P2]_4_ in solution, although [C2B2P2]_2_ is required for stable binding to the three consecutive CP2c half-sites or to the two staggered CP2c motifs. As a plausible model, we suggest a scenario where stable CBP complexes interact with each other to form moderately stable dimeric and tetrameric complexes in solution, owing to CP2c–CP2c, CP2c–CP2b, or CP2b–CP2b interactions between the CBPs. For binding to the three consecutive CP2c half-sites, each [C2B2P2]_2_ binds to a CP2c binding motif (i.e., a pair of CP2c half-sites 1 and 2), forming an unstable DNA–CBP complex due to the capability of one CP2c to bind to each CP2c half-site (since CP2b in the complex does not have a DBD) unless another CBP unstably binds to the other CP2c-binding motif sharing the central CP2c half-site (i.e., a pair of CP2c half site 2 and 3) in the opposite side of DNA, allowing stable DNA–[C2B2P2]_2_ complex formation owing to the CP2c dimer binding to the central CP2c half site. Similarly, two CBPs bind to two staggered CP2c binding motifs by forming two unstable DNA–CBP complexes (via two consecutive CP2c half-sites) in the opposite side of DNA in a way similar to that of three consecutive CP2c half-sites, but stabilization of two CBPs to DNA occurs not by sharing the CP2c half-site in the middle, but rather by CP2c–CP2c interaction in between CBPs. However, the detailed mechanisms underlying these scenarios require additional molecular and structural studies.

**Figure 9:**
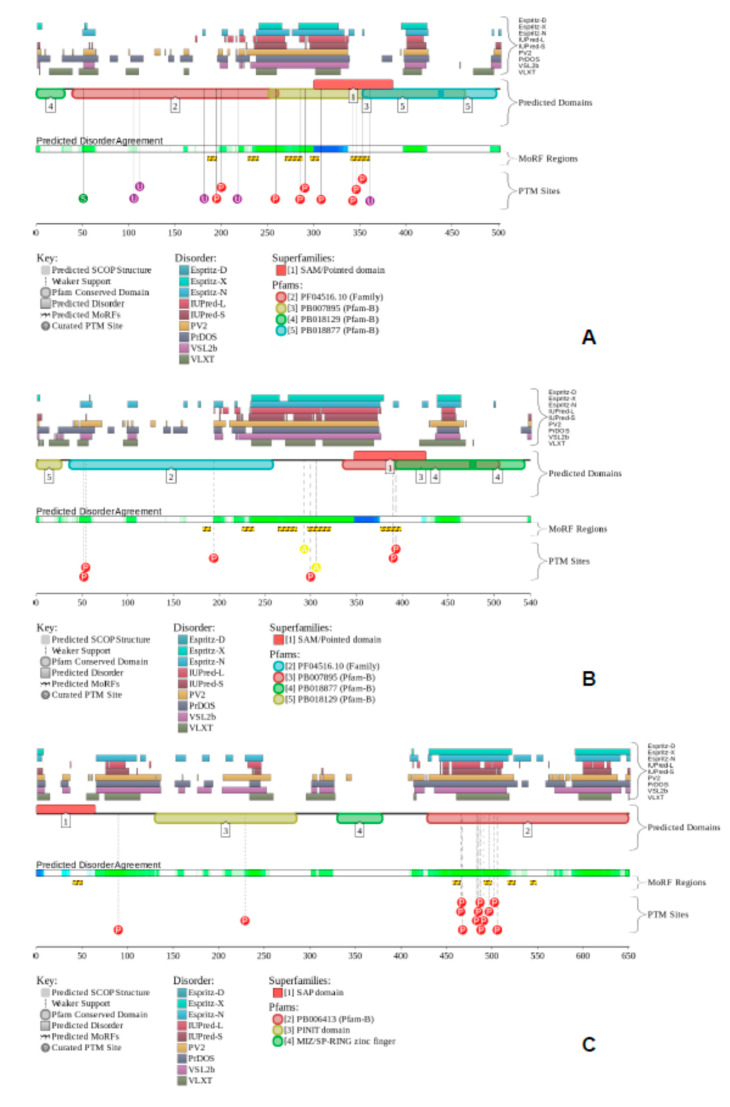
Intrinsic disorder predisposition of mouse CP2c (UniProt ID: Q9ERA0; (**A**)), CP2b (UniProt ID:Q811S7; (**B**)), and Pias1 (UniProt ID: O88907; (**C**)). Functional disorder profiles were generated by the computational platform D2P2 (http://d2p2.pro/, accessed on 3 June 2022), which is a database of disordered protein predictions [40]. In addition to disorder predispositions of query proteins evaluated by IUPred [41], PONDR^®^ VLXT [42], PrDOS [43], PONDR^®^ VSL2B [44,45], PV2 [40], and ESpritz [46], this database is further supplemented by data concerning the location of functional domains, various curated posttranslational modifications, and predicted disorder-based protein binding sites, known as molecular recognition features, MoRFs.

Since TFs regulate gene expression by binding DNA sequences recognized by their DBDs, yet DBD-recognized DNA motifs are short and highly abundant in genomes, TFs must bind to a specific subset of motif-containing sites rapidly upon activation to cope with cellular needs. Since the rate at which a TF encounters a binding site depends on the effective interaction volume, corresponding principally to the size of the site, several models of TF motion within the complex nuclear environment have been proposed, including 3D Brownian diffusion in the nucleoplasm, 1D sliding along the DNA, facilitated by nonspecific TF DNA binding, intersegmental transfer, hopping, and intersegmental jumping [29,30,31,32]. In addition, mechanisms contributing to the TF target search beyond the core DBD-recognized motif are proposed, such as sequences flanking the core motif, DNA accessibility within the chromatin-packed eukaryotic genomes, cooperative binding by the formation of multi-TF complexes, and a role for long intrinsically disordered regions (IDRs) outside the DBDs in the TF [39]. In line with these observations, Figure 9 represents disorder profiles generated for mouse CP2c (UniProt ID: O88907), CP2b (UniProt ID: Q811S7), and Pias1 (UniProt ID: O54714) by the computational platform D^2^P^2^ (http://d2p2.pro/, accessed on 3 June 2022), which is a database of disordered protein predictions [40]. It includes data generated by a set of commonly utilized disorder predictors and their variants: PONDR^®^ VLXT, PONDR^®^ VSL2b, PrDOS, PV2, Espritz, and IUPred. These tools were used to pre-calculate the disorder predisposition of 10,429,761 proteins from 1765 complete proteomes. The output of D^2^P^2^ for a target protein is further enhanced by showing the location of functional domains, predicted disorder-based protein binding sites, known as molecular recognition features, MoRFs, which are disordered sub-regions capable of binding-induced folding, and sites of various post-translational modifications [40]. Analysis of Figure 9 shows that all three proteins related to the CP2c complexes contain noticeable levels of intrinsic disorder. Furthermore, they contain numerous MoRFs, which can define their capability to be engaged in multiple protein–protein interactions.

**Figure 10:**
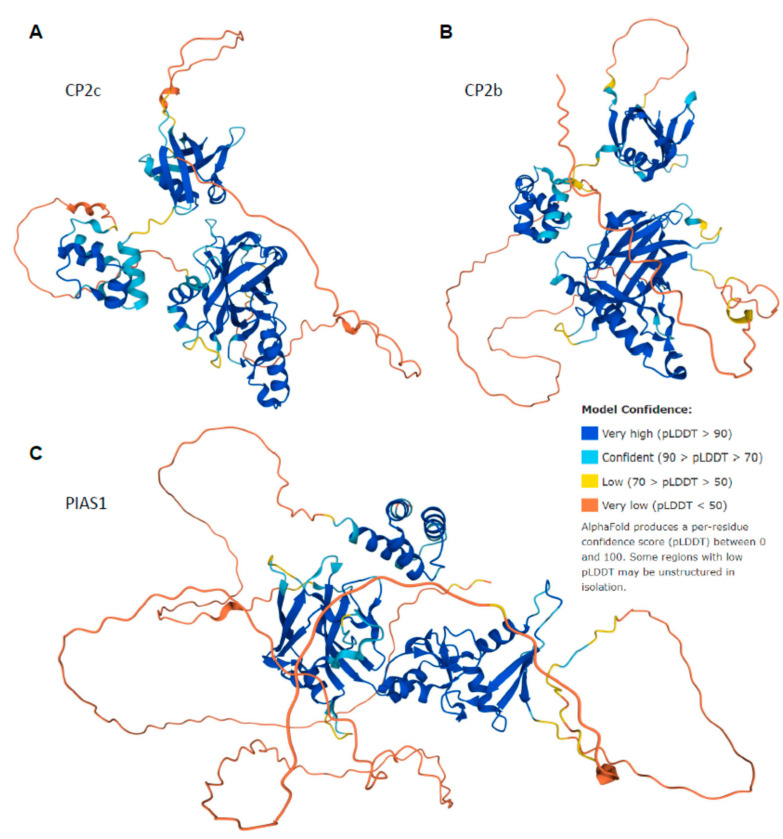
3D structures of CP2c (**A**), CP2b (**B**), and Pias1 (**C**) modeled by AlphaFold. Note that in addition to globular domains (shown by blue and cyan colors) these proteins contain long regions, whose structures are predicted with low or very low confidence (yellow and orange segments), which might be unstructured in isolation.

The idea of high disorder content in CP2c, CP2b, and Pias1 is further supported by Figure 10, representing 3D structures of these proteins modeled by AlphaFold {PMID: 34265844}. According to this analysis, CP2c is expected to have three globular domains (residues 66–281 (which is included in the Grh/CP2 DNA-binding domain, residues 63–300), 323–383, and 389–502) connected by flexible loops (see Figure 10A). Furthermore, N-terminal residues 1–65 and the 397–424 loop in the C-terminal domain are expected to be disordered as well, which correlates with the results of disorder prediction showing a high prevalence of disorder within the first 66 residues of this protein and in the 395–424 region. Curiously, the longest predicted disordered region in this protein (residues 228–332) includes a C-terminal part of the DNA-binding domain (residues 228–281), an N-terminal region of the middle globular domain (residues 323–332), and a long loop connecting these domains (residues 282–322). Figure 10B shows that CP2b is characterized by a similar structural organization containing three globular domains (residues 64–273, 360–422, and 427–540) and three long disordered regions (residues 1–63, 439–461, and 274–361). The first ordered region represents the core of the Grh/CP2 DNA-binding domain of this protein. A highly disordered N-tail (residues 1–40) is responsible for transcription activation, whereas the 274–309 region, which is a part of the disordered loop connecting first two globular domains and is predicted to overlap with two MoRFs (residues 264–284 and 296–321), plays a role in the erythroid-specific transcriptional activation.

Finally, Figure 10C shows that although Pias1 also has three globular domains (residues 1–64, 132–287, and 290–415) and two very long disordered regions (residues 65–131 and 416–651), their amino acid sequence positioning is very different from those of CP2c and CP2b. The first two globular domains of Pias1 include the functional domains SAP (residues 11–45, a putative DNA-binding motif involved in chromosomal organization named after SAF-A/B, Acinus, and PIAS, three proteins known to contain it) and PINIT (residues 124–288). Furthermore, the disordered C-tail includes a SUMO1-binding motif (residues 462–473) and a region with four NTLS repeats (residues 620–615). Altogether, Figure 10 shows that due to their highly flexible structural organization, these three proteins might have multiple modes to form various complexes. Furthermore, it is expected that the conformational ensembles of these proteins could be extremely sensitive to the peculiarities of their cellular environments, suggesting that even subtle changes in the physiological conditions can generate a strong conformational response, leading to very different outputs, such as formation of the homotetrameric CP2c complex (tCP2c) or heterohexameric complex (CBP) containing CP2c, CP2b, and Pias1.

Although the cell nucleus contains a mixture of macromolecules with chromatin, sophisticated and precise gene regulation must somehow take place in this environment for cellular homeostasis in normal cells. A liquid–liquid phase separation hypothesis, in which multi-molecular assemblies would form by phase separation bridging enhancers and promoters allowing gene activation [47], may help to explain how chromatin is organized in the nucleus and implies a spatiotemporal concentration of biomolecules, altering their localization and activities in cells. Biomolecular condensates originating as a result of highly controlled biological liquid–liquid phase transitions can fuse, coalesce, and drip, which are typical properties of liquid assemblies [48]. The macromolecular multiprotein complexes known as promyelocytic leukemia nuclear bodies (PML NBs) are an archetype for nuclear membrane-less organelles. Eukaryotic TFs usually contain long intrinsically disordered regions (IDRs) outside of the DBDs that are proposed to recognize specific DNA sequences or geometrical features like chromatin, or be recruited by other DNA-binding proteins [49,50]. Importantly, IDRs are also proposed to contribute to the formation of phase-separated condensates [27,51,52,53,54,55,56,57,58], with proteomes of various human membrane-less organelles being systematically enriched in disordered proteins that play a number of functional roles in the biogenesis of these organelles [59]. Thus, chromatin interactions with nuclear bodies are accepted to regulate genome function [60]. Within this context, it is suggested that both CP2c complexes collaborate with each other for efficient finding of DNA target sites within physiologically relevant timescales by facilitating intersegmental transfer of CBP multimers between sections of nuclear DNA via an antenna effect of tCP2c. It is also suggested that nuclear [C2B2P2]_4_ exerts a crucial role in a facilitated intersegmental transfer mechanism between two CP2c-binding DNA sites, where a DNA-bound [C2B2P2]_4_ interacts with another DNA target, nearby or in proximal localization within a nuclear condensate, via protein–DNA interaction forming a DNA loop or an inter-strand joining.

## 4. Materials and Methods

### 4.1. Cell Culture

The murine erythroleukemia (MEL DS19, donation from Dr. Mark Groudine), human embryonic kidney (293T; ATCC no. CRL-3216), and human breast cancer (the LM1 line of MDA-MB-231, donation from Prof. Su-Jae Lee) cell lines were maintained in high-glucose Dulbecco’s Modified Eagle’s Medium (DMEM; HyClone, South Logan, UT, USA, SH30243.01) supplemented with 10% fetal bovine serum (FBS; Cellsera, Rutherford, NSW, Australia, AU-FBS/PG), 100 units/mL penicillin (Sigma-Aldrich, Saint Louis, MO, USA, P3032), and 100 µg/mL streptomycin (Sigma-Aldrich, S9137). All cell lines were cultured at 37 °C, 5% CO_2_ in an incubator. All cell transfections (for the transient transfection of plasmid) were performed via Effectene reagent (Qiagen, San Diego, CA, USA, 301425). For erythroid terminal differentiation experiments, MEL cell lines were induced by supplementing the medium with the chemical inducer hexamethylene-bisacetamide (5 mM) (HMBA, Sigma-Aldrich, H4663).

### 4.2. Plasmid Construction

The DNA sequences, containing a CP2c-binding sequence or a mutated CP2c-binding sequence, were synthesized as oligonucleotides (listed in Table S1) for the purposed transactivation activity check by the luciferase assay. Each of the oligonucleotides was mixed and annealed in a TEN buffer (20 mM Tris-HCl, pH 8.0, 100 mM NaCl, and 1 mM EDTA). The oligonucleotides for mouse Gata1 proximal enhancer sequence WT, Mut1/3, Mut 2/4, and Mut 1–4 were cloned into the Xho I and Hind III digested pGL3-promoter vector.

### 4.3. Chromatin Immunoprecipitation-Quantitative PCR (ChIP-qPCR)

Uninduced MEL cells and MEL cells induced by treatment of HMBA for 3 days were used for ChIP analysis. Harvested cells (1 × 10^7^) were crosslinked by rotating with 1% formaldehyde (Sigma-Aldrich, 252549) in PBS for 10 min at room temperature. Crosslinking was quenched by rotating with 125 mM glycine (Sigma-Aldrich, G4392) in PBS for 5 min at room temperature. Cells were rinsed twice with ice-cold PBS and lysed with 250 μL of the lysis buffer (10 mM Tris–HCl, pH 8.0, 10 mM NaCl, 100 mM CaCl_2_, and 0.1% NP-40). Genomic DNA was decomposed by enzyme digestion for 30 min at 37 °C using 10 U/μL Micrococcus Nuclease (Sigma Aldrich, N3755) and sonication for 4 periods of 10 s pulse on ice using a sonicator (Hielscher, Warthestrasse, Teltow, Germany, UP200H) to generate 200–300 bp DNA fragments. After centrifugation at 13,000 rpm for 10 min at 4 °C, the supernatant was pre-cleared with 50 μL Protein A/G agarose beads (Thermo Fisher, Waltham, MA, USA, 20421). Then, the pre-cleared chromatin extracts were incubated overnight at 4 °C with 100 μL Protein A/G agarose beads, pre-incubated with 3 μg of the appropriate ChIP-grade antibodies or IgG for at least 3 h. The beads were washed twice with 500 μL ChIP washing buffer 1 (20 mM Tris–HCl, pH 8.0, 150 mM NaCl, 2 mM EDTA, 0.1% SDS, and 1% Triton X-100), once with 500 μL ChIP washing buffer 2 (10 mM Tris–HCl, pH 8.0, 250 mM LiCl, 1 mM EDTA, 1% SDS, and 1% NP-40), and finally twice with 500 μL TE (10 mM Tris pH 8.0 and 1 mM EDTA) sequentially. The complex was eluted by rotating with 200 μL freshly prepared elution buffer (100 mM NaHCO_3_ and 1% SDS) for 30 min at room temperature. Then the reverse crosslinking was carried out by adding 250 mM NaCl and incubating overnight at 65 °C. DNA was treated with RNase A (0.2 mg/mL final) and proteinase K (0.2 mg/mL final) for 2 h at 37 °C. Then, DNA was purified by phenol/chloroform extraction and ethanol precipitation. The pellets were dissolved in 100 μL TE buffer for qPCR. qPCR assays were performed using SYBR green (TaKaRa, Kusatsu, Japan, RR420A) with the specific primers listed in Table S1. The data were normalized to the input DNA and enrichment was calculated by fold excess over ChIP performed with specific IgG as background signal. All assays were conducted in duplicate. Primary antibodies used for ChIP were CP2c (Abcam, Cambridge, MA, USA, ab42973; BD bioscience, San Jose, CA, USA, 610818), CP2b (custom antibody obtained from Peptron), and Pias1 (Abcam, ab32219).

### 4.4. Dual Luciferase Assay

A firefly luciferase reporter construct containing WT or mutated CP2c binding sites from the *GATA1* proximal enhancer region and a control renilla luciferase reporter construct containing a CMV promoter region were employed. 293T cells were transiently transfected with 400 ng of DNA, including the luciferase reporter constructs and various combinations of CP2c (WT and point mutants), CP2b, and Pias1 expression vectors using Effectene in 12-well tissue culture plates. The transfection ratio of the firefly luciferase vector and the control renilla luciferase vector was 5:1. Cells were harvested 48 h after transfection with 100 µL passive lysis buffer (Promega, Madison, WI, USA, E1941). To estimate luciferase activity, 20 µL aliquots of each lysate were used for quantification using the dual-luciferase reporter assay system (Promega, E1910) on the Lumat LB9501 Luminometer (Berthold, Bad Wildbad, Germany). Firefly luciferase activity (Fluc) was normalized against renilla luciferase activity (Rluc) and the data were represented as the ratio of firefly to renilla luciferase activity (Fluc/Rluc).

### 4.5. Cell Extract Preparation

Cell extracts were prepared according to Kim et al. [24]. Briefly, cytosolic extracts were prepared using the lysis buffer (50 mM Tris-HCl, pH 7.4, 150 mM NaCl, 1 mM EDTA, 0.1% triton X-100, and 1 mM PMSF) for general Western blot. Nuclear extracts were prepared from MDA-MB-231 cells and MEL cells (uninduced or induced with 5 mM HMBA) for determining CP2c-containing complexes. Nuclear extracts were prepared using cell lysis buffer A (50 mM Tris-HCl, pH 8.0, 10 mM NaCl, 0.2% NP-40, 10 mM EDTA, and protease inhibitor cocktail) and nuclei lysis buffer B (50 mM Tris-HCl, pH 8.0, 10 mM EDTA, 1% SDS, and protease inhibitor cocktail). Each of the cytosolic and nuclear extracts used for DSP XL-WB was derived from an equal number of cells.

### 4.6. DNA Immunoprecipitation (DIP) Assay

Each of the oligonucleotides was mixed and annealed in TEN buffer. For the radio-labeling of the DNA probe, the annealed DNA was incubated with a mixture of dATP, dGTP, dTTP, [α^32^P]-dCTP, and Klenow enzyme in reaction buffer (5 mM NaCl, 1 mM Tris HCl, 1mM MgCl_2_, and 0.1 mM DTT) for 30 min at 25 °C, and stopped by incubation with EDTA (10 mM final) for 20 min at 75 °C. The radio-labeled DNA probe was purified by applying the reaction mixture to ProbeQuant G-50 micro columns (GE healthcare, Chicago, IL, USA, GE28-9034-08). Nuclear extracts prepared from transfected 293T cells were incubated with an [α^32^P]-labeled DNA probe in binding buffer (4% glycerol, 10 mM Tris-HCl, pH 7.4, 1 mM DTT, 1 mM EDTA, and 0.1% NP-40) for 15 min at room temperature. For immunoprecipitation, precleared extracts were incubated overnight at 4 °C with 1 μg of the following primary antibodies: CP2c, CP2b, and Pias1. Then 50 µL Protein A/G agarose beads were added to the mixture and incubated for another 3 h at 4 °C. The precipitated complexes were washed three times with wash buffer (50 mM tris-HCl, pH7.4, 150 mM NaCl, 1 mM EDTA, and 1 mM PMSF). The labeled DNA probes were eluted from the precipitated DNA–protein complex with elution buffer (50 mM Tris-HCl, pH7.4, 10 mM EDTA, and 1% SDS) for 1 h at 65 °C. The radioactivity of the eluted probe was measured by scintillation counting.

### 4.7. Western Blot

The pull-down or immunoprecipitated samples were separated by SDS-PAGE and electroblotted onto polyvinylidene difluoride (PVDF) membranes (GE healthcare, 10600069). Membranes were blocked with 5% BSA in a solution of 0.1% tween 20 and incubated overnight at 4 °C with appropriate dilutions of the HRP-conjugated streptavidin (Thermo, 89880D) or the following primary antibodies: CP2c (Abcam, ab155238; BD biosciences 610818), CP2b (Santa cruz, Dallas, TX, USA, sc-81310; Rabbit-CP2b), Pias1 (Abcam, ab32219; Santa cruz, sc-365217), p53 (Abcam, ab131442; Santa cruz, sc-216), HA (Abcam, ab49969), Flag (Sigma-Aldrich, F1804), and EGFP (Abcam, ab5449). The blots were incubated for 1 h at room temperature with the following respective HRP-conjugated secondary antibodies: mouse IgG HRP (Thermo Fisher, 31430, 1:10,000), goat IgG HRP (Thermo Fisher, 811620, 1:10,000), and rabbit IgG HRP (Abcam, ab6802 1:20,000). Polyclonal ACTB antibody was used as a loading control for immunoblotting. Proteins were visualized by chemiluminescence using an ECL system (GE healthcare, RPN2106). Relative amounts of proteins were quantified using the Image J (ver. 1.51) program.

### 4.8. Probe Titration Assay

For analyzing the DNA binding affinity of each CP2c-containing complex, nuclear lysates from transfected 293T cells were mixed with biotin-conjugated double-stranded DNA probes in a concentration dependent manner (0~100 nM). Samples with various combinations were incubated for 2 h at room temperature with Streptavidin-Sepharose beads (Invitrogen, Waltham, MA, USA, 15942-050). Pull down samples were washed with lysis buffer once. For immunoblotting, protein loading samples were prepared by boiling in 2Χ SDS-PAGE sample loading buffer. Proteins were visualized by chemiluminescence using an ECL system (GE healthcare, RPN2106). Relative amounts of proteins were quantified using the Image J (ver. 1.51) program. The proportion of protein bound to DNA was derived by calculating the band intensity of Western blot as output versus input. Relative amounts of CP2c in the tCP2c were obtained by subtracting the amounts of CP2b or Pias1 from the total amounts of CP2c, assuming that the amounts of CP2c in the CBP were the same as those of CP2b or Pias1 [23].

### 4.9. DSP [Dithiobis(Succinimidyl Propionate)] Crosslinking and Western Blot (DSP XL-WB)

For determination of DNA–CP2c-containing complexes, biotin-conjugated double-stranded DNA probes (WT or mutant probe of the *Hba-a2* promoter and *Gata1* enhancer) were crosslinked with nuclear lysates from MDA-MB-231 cells using a DSP crosslinker (final 2 mM) for 30 min at room temperature. Crosslinking was terminated by adding Tris-HCl (final 20 mM, pH 8.0) at room temperature. Various samples were incubated overnight at 4 °C with the Streptavidin-Sepharose beads (Invitrogen, 15942-050). Pull-down samples were washed with lysis buffer three times.

For identification of DNA-free transcription factor complexes, nuclear or cytosolic lysates were prepared from the same number of cells. Each nuclear or cytosolic lysate was treated with Benzonase to exclude the influence of DNA and was crosslinked using a DSP crosslinker (final 2 mM) for 30 min at room temperature. Crosslinking was terminated by adding Tris-HCl (final 20 mM, pH 8.0) at room temperature. Various samples were incubated overnight at 4 °C with the appropriate antibodies for co-immunoprecipitation. For immunoprecipitation, precleared extracts were incubated overnight at 4 °C with 10 μL Protein A/G agarose beads, pre-incubated with 2 μg of the following appropriate primary antibodies: CP2c (Abcam, ab155238; BD biosciences 610818), CP2b (Santa cruz, sc-81310; Rabbit-CP2b), Pias1 (Abcam, ab32219; Santa cruz, sc-365217), p53 (Abcam, ab131442; Santa cruz, sc-216), HA (Abcam, ab49969), Flag (Sigma-Aldrich, F1804), and EGFP (Abcam, ab5449). The immune complexes were washed 3 times with lysis buffer, and the bound proteins were eluted with 2× bed volume of 0.2 M glycine buffer, followed by neutralization with an equal volume of 1 M Tris-HCl, pH 8.0. For Flag tag immunoprecipitation, precleared extracts were incubated with 2 μL of Flag-M2 beads (Sigma-Aldrich, A2220) by rotating overnight at 4 °C. The immune complexes were washed 3 times with lysis buffer, and the bound proteins were eluted with 100 µg/mL Flag peptide (Sigma-Aldrich, F4799).

Half of each sample (pull-down sample and immunoprecipitated sample) was analyzed together with the crosslinker cleaved by adding DTT (final 50 mM). For immunoblotting, protein loading samples were prepared by boiling in 2Χ SDS-PAGE sample loading buffer. The pull-down or immunoprecipitated samples were separated by SDS-PAGE and electroblotted onto PVDF membranes. Membranes were blocked with 5% BSA in a solution of 0.1% Tween 20 and incubated overnight at 4 °C with appropriate dilutions of the HRP-conjugated streptavidin or primary antibodies (listed in Section 4.7). Deprobing was performed by reacting with strip buffer (52.6 mM Tris pH6.8, 2% SDS, 100 mM beta-mercaptoethanol) at 65 °C for 30 min, followed by washing with PBS-T for 30 min. After deprobing, membranes were blocked with 5% BSA in a solution of 0.1% Tween 20 and incubated overnight at 4 °C with appropriate dilutions of the HRP-conjugated streptavidin or primary antibodies.

### 4.10. Quantification and Statistical Analysis

Data are presented as mean ± standard error. The sample size for each experiment, n, was included in the Results section and the associated figure legend. Throughout the text, the difference between two subsets of data was considered statistically significant if the one-tailed Student’s t test gave a significance level (*p* value) less than 0.05. Statistical analysis was performed in GraphPad Prism 6.

## 5. Conclusions

Our findings about the structural and functional aspects of cellular CP2c complexes will provide fundamental and crucial clues for developing inhibitors for both basic research and clinical applications. In addition, although pharmacologic inhibition of a TF or cofactor that acts widely on genes throughout the genome can exert highly selective effects on cancer control due to its oncogenic addiction in cancer cells, only very few have been successfully advanced by coordinated efforts in drug discovery, in part due to lacking knowledge of the detailed structural and functional nature in cells. Thus, our methodologies uncovering several unprecedented findings about stoichiometries, DNA binding targets, and regulation of nuclear levels of CP2c TF complexes will provide a paradigm for studies in other important oncogenic TFs, leading to the successful drug development.

## Data Availability

Not applicable.

## Supplementary Materials

The following supporting information can be downloaded at: https://www.mdpi.com/article/10.3390/ijms23126369/s1.

## Abbreviations

CBP	Heterohexameric CP2c, CP2b, and PIAS1 complex
ChIP-qPCR	Chromatin immunoprecipitation-quantitative PCR
DIP	DNA co-immunoprecipitation
DSP	Dithiobis (succinimidyl propionate)
DSP XL-WB	DSP crosslinking and Western blot
EMT	Epithelial–mesenchymal transition
FBS	Fetal bovine serum
HCC	Hepatocellular carcinoma
HIV/AIDS	Human immunodeficiency virus infection and acquired immunodeficiency syndrome
IDRs	Intrinsically disordered regions
MoRFs	Molecular recognition features
PAGE	Polyacrylamide gel electrophoresis
SDS	Sodium dodecyl sulfate
tCP2c	Homotetrameric CP2c complex
TFs	Transcription factors
WT	Wild type
