# Supplementary material for: Structural and Functional Insights into CP2c Transcription Factor Complexes
Source: Int J Mol Sci. 2022 Jun 7;23(12):6369. doi: 10.3390/ijms23126369 (PMC9223585; doi:10.3390/ijms23126369)
Supplement: Supplementary file 1 [file ijms-23-06369-s001.zip › ijms-1740285-supplementary.pdf]

Supplementary Materials

Structural and functional insights into CP2c transcription factor complexes

Seung Han Son[1,§], Min Young Kim[1,§], Eunbi Jo[1], Vladimir N. Uversky[2,*], Chul Geun Kim[1,3,*]

[1] Department of Life Science and Research Institute for Natural Sciences, College of Natural Sciences, Hanyang University, Seoul 04763, Korea; imsangok12@naver.com (S.H.S.); choeunbi0324@naver.com (E.J.); 5718my@naver.com (M.Y.K); cgkim@hanyang.ac.kr (C.G.K.)

[2] Department of Molecular Medicine, Morsani College of Medicine, University of South Florida, Tampa, Florida 33612, USA; vuversky@usf.edu (V.N.U.)

[3] CGK Biopharma Co. Ltd., 222 Wangshipri-ro, Sungdong-gu, Seoul 04763, Korea; cgkim@hanyang.ac.kr (C.G.K.)

*Correspondence: vuversky@usf.edu (V.N.U.); cgkim@hanyang.ac.kr (C.G.K.)

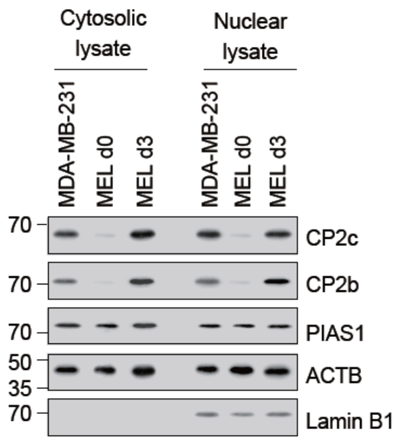

Figure S1. Endogenous CP2b exists in both nucleus and cytosol. Cytosolic lysate and nuclear lysate were subjected to western blots by using specific antibodies against CBP complex proteins. Lamin B1 was employed as a nuclear protein control.

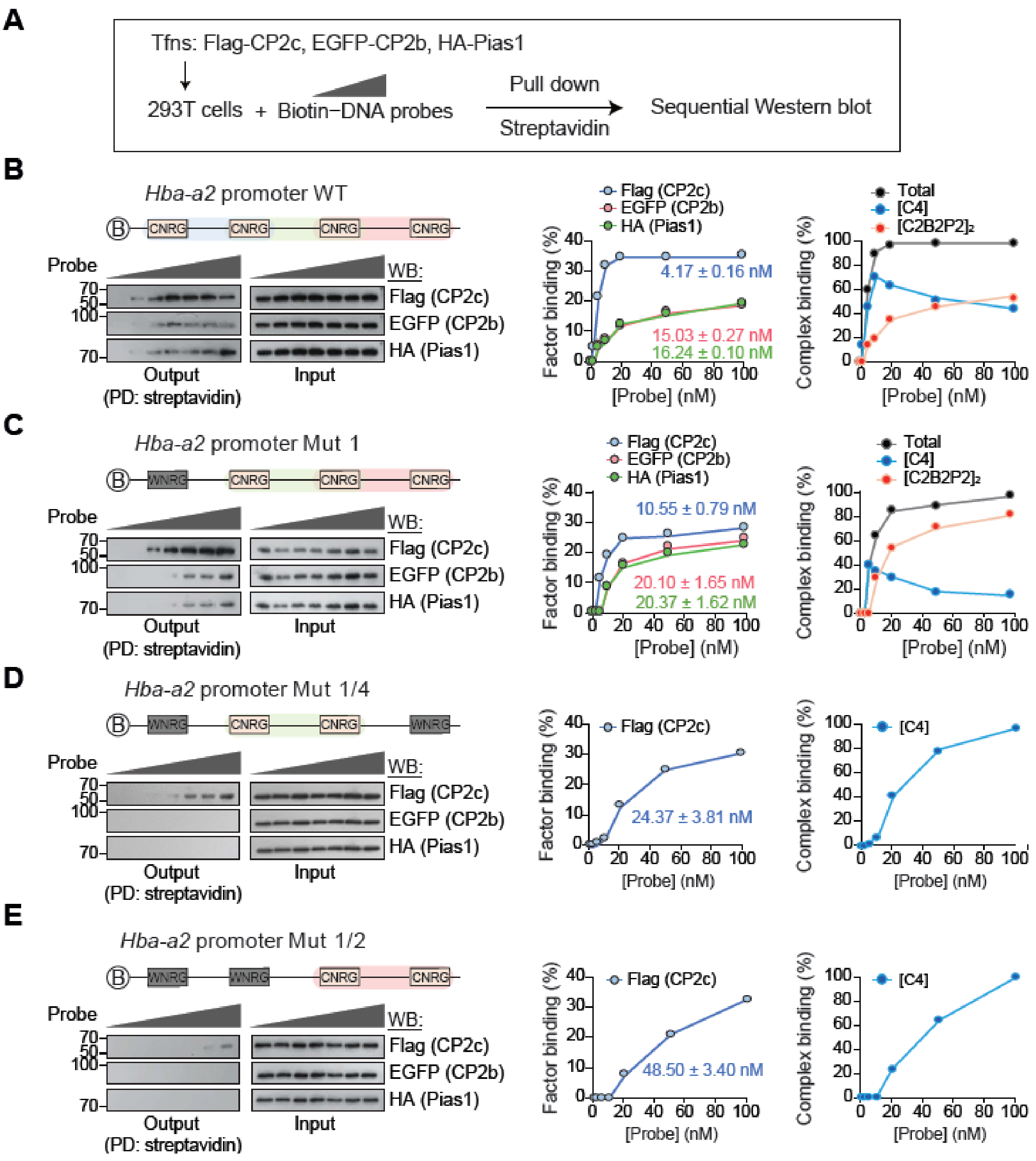

Figure S2. [C4] shows synergism to both [C4] and [C2B2P2]$_2$ for target DNA binding. (**A**) Experimental scheme to identify the binding efficiency of CP2c complex proteins *in vitro* by South-Western analysis. Immunoblots and quantified DNA binding efficiency with estimated Kd in the *Hba-a2* promoter probes with WT (**B**), Mut 1 (**C**), Mut 1/4 (**D**), and Mut 1/2 (**E**).

Table S1. Oligonucleotides

name	sequence
**for Plasmid construction**	
CP2 BS probe_F	5'- GCT AGC GAT CCC AAG TTT TAC TCG GTA GAG CAA GCA CAA ACC AGG-3'
CP2 BS probe_R	5'- CTC GAG CCT GGT TTG TGC TTG CTC TAC CGA GTA AAA CTT GGG ATC-3'
Gata1 WT enhancer F	5' - GCT AGC CGA GTC CAT CTG ATA AGA CTT ATC TGC TGC CCC AGA GCA GGC CAG AGC TGG CGT AAG CCC CAG -3'
Gata1 WT enhancer R	5' - CTC GAG CTG GGG CTT ACG CCA GCT CTG GCC TGC TCT GGG GCA GCA GAT AAG TCT TAT CAG ATG GAC TCG -3'
Gata1 Mut 1/3 enhancer F	5' -GCT AGC CGA GTC CAT CTG ATA AGA CTT ATC TGC TGC CAC AGA GCA GGT AAG AGC TGG CGT AAG CCC CAG - 3'
Gata1 Mut 1/3 enhancer R	5' - CTC GAG CTG GGG CTT ACG CCA GCT CTT ACC TGC TCT GTG GCA GCA GAT AAG TCT TAT CAG ATG GAC TCG - 3'
Gata1 Mut 2/4 enhancer F	5' - GCT AGC CGA GTC CAT CTG ATA AGA CTT ATC AGC AGC CCC AGA TTG AGC CAG ATT GAG CGT AAG CCC CAG -3'
Gata1 Mut 2/4 enhancer R	5' - CTC GAG CTG GGG CTT ACG CTC AAT CTG GCT CAA TCT GGG GCA GCA GAT AAG TCT TAT CAG ATG GAC TCG - 3'
Gata1 Mut 1-4 enhancer F	5' - GCT AGC CGA GTC CAT CTG ATA AGA CTT ATC TGC TGC CAC AGA TTG AGT AAG ATT GAG CGT AAG CCC CAG - 3'
Gata1 Mut 1-4 enhancer R	5' - CTC GAG CTG GGG CTT ACG CTC AAT CTT ACT CAA TCT GTG GCA GCA GAT AAG TCT TAT CAG ATG GAC TCG - 3'
**for Chromatin immunoprecipitation quantitative PCR (ChIP-qPCR)**	
MRE HS26-2_F	5'-CCA CTG TGG GAT CTA TGG AG-3'
MRE HS26-2_R	5'-CTT TCT GAC TCT GTA CCC CC-3'
MRE HS21-2_F	5'-GCA CCT ATG CTC ACC TAT CT-3'
MRE HS21-2_R	5'-CTT CCC TCA CTG AGA ATG TCT-3'
MRE HS21-4_F	5'-TGG AGG CAG CTG GAA CAG AA-3'
MRE HS21-4_R	5'-GCC TGG AAG TCC TGT GGT A-3'
Hba-a2 upstream_F	5'-GTC ACC AAA ACC AGC TCA GA-3'
Hba-a2 upstream_R	5'-GCC AGA CTC CTC CTT CAG AC-3'
Hba-a2 promoter_F	5′- GAT CCC AAG TTT TAC TCG GTA GAG CAA GCA CAA ACC AGG-3′
Hba-a2 promoter_R	5′- GAT CCC TGG TTT GTG CTT GCT CTA CCG AGT AAA ACT TGG -3′
LCR HS2_F	5'-AGG GTG TGT GGC CAG ATG TT-3'
LCR HS2_R	5'-ACC CAG ATA GCA CTG ATC AGT CAC-3'
Hbb-b1 promoter_F	5'-GCT TCT GAC ATA GTT GTG TTG ACT CA-3'
Hbb-b1 promoter_R	5'-CAG CAG CCT TCT CAG CAT CA-3'
Gata1 enhancer F	5'-CGA GTC CAT CTG ATA AGA CT-3'
Gata1 enhancer R	5'-CTG CAG GCC ACC TCT ATG AA-3'
Nfe2_promoter_F	5'-GGT AAA GGT CCA GTG TCT CC-3'
Nfe2_promoter_R	5'-GTG ATA GCA ACC CTT CCC TC-3'
**for DSP crosslinking (western blot and titration assay)**	
CP2 WT BS probe_F (Bio)	5'-/5Biosg/GAT CCC AAG TTT TAC TCG GTA GAG CAA GCA CAA ACC AGG-3'
CP2 WT BS probe_R (Bio)	5'-/5Biosg/CCT GGT TTG TGC TTG CTC TAC CGA GTA AAA CTT GGG ATC-3'
CP2 Mut1 BS probe_F (Bio)	5'-/5Biosg/GAT CCA AAG TTT TAC TCG GTA GAG CAA GCA CAA ACC AGG-3'
CP2 Mut1 BS probe_R (Bio)	5'-/5Biosg/CCT GGT TTG TGC TTG CTC TAC CGA GTA AAA CTT TGG ATC-3'
CP2 Mut1/2 BS probe_F (Bio)	5'-/5Biosg/GAT CCA AAG TTT TAT TCG GTA GAG CAA GCA CAA ACC AGG-3'
CP2 Mut1/2 BS probe_R (Bio)	5'-/5Biosg/CCT GGT TTG TGC TTG CTC TAC CGA ATA AAA CTT TGG ATC-3'
CP2 Mut1/4 BS probe_F (Bio)	5'-/5Biosg/GAT CCA AAG TTT TAC TCG GTA GAG CAA GCA CAA ATC AGG-3'
CP2 Mut1/4 BS probe_R (Bio)	5'-/5Biosg/CCT GAT TTG TGC TTG CTC TAC CGA GTA AAA CTT TGG ATC-3'
CP2 Mut4 BS probe_F (Bio)	5'-/5Biosg/GAT CCC AAG TTT TAC TCG GTA GAG CAA GCA CAA ATC AGG-3'
CP2 Mut4 BS probe_R (Bio)	5'-/5Biosg/CCT GAT TTG TGC TTG CTC TAC CGA GTA AAA CTT GGG ATC-3'
CP2 Mut3/4 BS probe_F (Bio)	5'-/5Biosg/GAT CCC AAG TTT TAC TCG GTA GAG TAA GCA CAA ATC AGG-3'
CP2 Mut3/4 BS probe_R (Bio)	5'-/5Biosg/CCT GAT TTG TGC TTA CTC TAC CGA GTA AAA CTT GGG ATC-3'
Gata WT enhancer F (Bio)	5'-/5Biosg/CGA GTC CAT CTG ATA AGA CTT ATC TGC TGC CCC AGA GCA GGC CAG AGC TGG CGT AAG CCC CAG-3'
Gata WT enhancer R (Bio)	5'-/5Biosg/CTG GGG CTT ACG CCA GCT CTG GCC TGC TCT GGG GCA GCA GAT AAG TCT TAT CAG ATG GAC TCG-3'
Gata Mut1/3 enhancer F (Bio)	5'-/5Biosg/CGA GTC CAT CTG ATA AGA CTT ATC TGC TGC CAC AGA GCA GGT AAG AGC TGG CGT AAG CCC CAG-3'
Gata Mut1/3 enhancer R (Bio)	5'-/5Biosg/CTG GGG CTT ACG CCA GCT CTT ACC TGC TCT GTG GCA GCA GAT AAG TCT TAT CAG ATG GAC TCG-3'
**for DNA-immunoprecipitation (DNA-IP)**	
CP2 WT BS probe_F	5'- GCT AGC GAT CCC AAG TTT TAC TCG GTA GAG CAA GCA CAA ACC AGG-3'
CP2 WT BS probe_R	5'- CTC GAG CCT GGT TTG TGC TTG CTC TAC CGA GTA AAA CTT GGG ATC-3'
CP2 Mut1 BS probe_F	5'- GCT AGC GAT CCA AAG TTT TAC TCG GTA GAG CAA GCA CAA ACC AGG-3'
CP2 Mut1 BS probe_R	5'- CTC GAG CCT GGT TTG TGC TTG CTC TAC CGA GTA AAA CTT TGG ATC-3'
CP2 Mut2 BS probe_F	5'- GCT AGC GAT CCC AAG TTT TAT TCG GTA GAG CAA GCA CAA ACC AGG-3'
CP2 Mut2 BS probe_R	5'- CTC GAG CCT GGT TTG TGC TTG CTC TAC CGA ATA AAA CTT GGG ATC-3'
CP2 Mut3 BS probe_F	5'- GCT AGC GAT CCC AAG TTT TAC TCG GTA GAG TAA GCA CAA ACC AGG-3'
CP2 Mut3 BS probe_R	5'- CTC GAG CCT GGT TTG TGC TTA CTC TAC CGA GTA AAA CTT GGG ATC-3'
CP2 Mut4 BS probe_F	5'- GCT AGC GAT CCC AAG TTT TAC TCG GTA GAG CAA GCA CAA ATC AGG-3'
CP2 Mut4 BS probe_R	5'- CTC GAG CCT GAT TTG TGC TTG CTC TAC CGA GTA AAA CTT GGG ATC-3'
CP2 Mut1/2 BS probe_F	5'- GCT AGC GAT CCA AAG TTT TAT TCG GTA GAG CAA GCA CAA ACC AGG-3'
CP2 Mut1/2 BS probe_R	5'- CTC GAG CCT GGT TTG TGC TTG CTC TAC CGA ATA AAA CTT TGG ATC-3'
CP2 Mut1/3 BS probe_F	5'- GCT AGC GAT CCA AAG TTT TAC TCG GTA GAG TAA GCA CAA ACC AGG-3'
CP2 Mut1/3 BS probe_R	5'- CTC GAG CCT GGT TTG TGC TTA CTC TAC CGA GTA AAA CTT TGG ATC-3'
CP2 Mut1/4 BS probe_F	5'- GCT AGC GAT CCA AAG TTT TAC TCG GTA GAG CAA GCA CAA ATC AGG-3'
CP2 Mut1/4 BS probe_R	5'- CTC GAG CCT GAT TTG TGC TTG CTC TAC CGA GTA AAA CTT TGG ATC-3'
CP2 Mut2/4 BS probe_F	5'- GCT AGC GAT CCC AAG TTT TAT TCG GTA GAG CAA GCA CAA ATC AGG-3'
CP2 Mut2/4 BS probe_R	5'- CTC GAG CCT GAT TTG TGC TTG CTC TAC CGA ATA AAA CTT GGG ATC-3'
CP2 Mut3/4 BS probe_F	5'- GCT AGC GAT CCC AAG TTT TAC TCG GTA GAG TAA GCA CAA ATC AGG-3'
CP2 Mut3/4 BS probe_R	5'- CTC GAG CCT GAT TTG TGC TTA CTC TAC CGA GTA AAA CTT GGG ATC-3'
Gata1 WT enhancer F	5' - GCT AGC CGA GTC CAT CTG ATA AGA CTT ATC TGC TGC CCC AGA GCA GGC CAG AGC TGG CGT AAG CCC CAG -3'
Gata1 WT enhancer R	5' - CTC GAG CTG GGG CTT ACG CCA GCT CTG GCC TGC TCT GGG GCA GCA GAT AAG TCT TAT CAG ATG GAC TCG -3'
Gata1 Mut1/3 enhancer F	5' -GCT AGC CGA GTC CAT CTG ATA AGA CTT ATC TGC TGC CAC AGA GCA GGT AAG AGC TGG CGT AAG CCC CAG - 3'
Gata1 Mut1/3 enhancer R	5' - CTC GAG CTG GGG CTT ACG CCA GCT CTT ACC TGC TCT GTG GCA GCA GAT AAG TCT TAT CAG ATG GAC TCG - 3'
Gata1 Mut2/4 enhancer F	5' - GCT AGC CGA GTC CAT CTG ATA AGA CTT ATC AGC AGC CCC AGA TTG AGC CAG ATT GAG CGT AAG CCC CAG -3'
Gata1 Mut2/4 enhancer R	5' - CTC GAG CTG GGG CTT ACG CTC AAT CTG GCT CAA TCT GGG GCA GCA GAT AAG TCT TAT CAG ATG GAC TCG - 3'
Gata1 Mut1-4 enhancer F	5' - GCT AGC CGA GTC CAT CTG ATA AGA CTT ATC TGC TGC CAC AGA TTG AGT AAG ATT GAG CGT AAG CCC CAG - 3'
Gata1 Mut1-4 enhancer R	5' - CTC GAG CTG GGG CTT ACG CTC AAT CTT ACT CAA TCT GTG GCA GCA GAT AAG TCT TAT CAG ATG GAC TCG - 3'
